# Ageing-related markers and risks of cancer and cardiovascular disease: a prospective study in the EPIC-Heidelberg cohort

**DOI:** 10.1007/s10654-021-00828-3

**Published:** 2021-12-22

**Authors:** Bernard Srour, Rudolf Kaaks, Theron Johnson, Lucas Cory Hynes, Tilman Kühn, Verena A. Katzke

**Affiliations:** 1 Division of Cancer Epidemiology, German Cancer Research Center, DKFZ, 69120 Heidelberg, Germany; 2 Institute for Global Food Security, Queen’s University, Belfast, Northern Ireland

**Keywords:** Ageing biomarkers, Cancer risk, Cardiovascular disease, Case-cohort, NT-proBNP

## Abstract

Biological age is an important risk factor for chronic diseases. We examined the associations between five markers of unhealthy ageing; Growth Differentiation Factor-15 (GDF-15), N-terminal pro-brain natriuretic peptide (NT-proBNP), glycated hemoglobin A1c (HbA1C), C-Reactive Protein (CRP) and cystatin-C; with risks of cancer and cardiovascular disease (CVD). We used a case-cohort design embedded in the EPIC-Heidelberg cohort, including a subcohort of 3792 participants along with 4867 incident cases of cancer and CVD. Hazard ratios (HRs) were computed and the strongest associations were used to build weighted multi-marker combinations, and their associations with cancer and CVD risks were tested. After adjusting for common confounders, we observed direct associations of GDF-15 with lung cancer risk, NT-proBNP with breast, prostate and colorectal cancers, HbA1C with lung, colorectal, and breast cancer risks, and CRP with lung and colorectal cancer risks. An inverse association was observed for GDF-15 and prostate cancer risk. We also found direct associations of all 5 markers with myocardial infarction (MI) risk, and of GDF-15, NT-proBNP, CRP and cystatin-C with stroke risk. A combination of the independently-associated markers showed a moderately strong association with the risks of cancer and CVD (HR_Q4-Q1_ ranged from 1.78[1.36, 2.34] for breast cancer, when combining NT-proBNP and HbA1C, to 2.87[2.15, 3.83] for MI when combining NT-proBNP, HbA1C, CRP and cystatin-C). This analysis suggests that combinations of biomarkers related to unhealthy ageing show strong associations with cancer risk, and corroborates published evidence on CVD risk. If confirmed in other studies, using these biomarkers could be useful for the identification of individuals at higher risk of age-related diseases.

**Supplementary Information:**

The online version contains supplementary material available at 10.1007/s10654-021-00828-3.

## Background

Non-communicable diseases (NCDs) are the number one cause of mortality worldwide. According to the Global Burden of Diseases (GBD), 73.4% of the deaths that occurred in 2017 were linked to NCDs, among which approximately 31% linked to cardiovascular diseases (CVD) and 29% to cancer [1]. Geographic and temporal variations in disease incidence rates worldwide [1] indicate that CVD and many common forms of cancer, especially those that have become frequent in more affluent societies, may share epidemiologic risk factors, in particular lifestyle factors such as smoking, diet, physical activity and energy balance, and various etiologic disease mechanisms triggered by these factors. Given these likely communalities in the etiology of multiple chronic disease types, there is interest in biological markers that may identify individuals at increased overall risk of prematurely developing any of these major diseases, in view of more personalized prevention strategies.

One area of particular interest in this regard relates to mechanisms of biological ageing, which may constitute a shared set of pathways increasing the susceptibility of developing a variety of diseases generally related to older age, including CVD, many frequent forms of cancer, and other degenerative diseases (“geroscience” hypothesis) [2–4]. Markers of biological ageing might be used for the prediction of overall chronic disease risk, and for providing novel insights into diseases etiology and their underlying biological pathways [5, 6]. In addition, there is increasing evidence that biological ageing processes can be specifically targeted and modulated through preventive and therapeutic interventions, including changes in dietary composition and energy restriction [7], increased physical activity [8] or drug treatments [9].

The biological process of ageing is related to a wide array of biological alterations. On the cellular level this includes increased genomic instability, epigenetic alterations, mitochondrial dysfunction, increased oxidative stress, loss of proteostasis, deregulated nutrient sensing, and cellular senescence [10]. On a more systemic level, ageing is characterized by losses and physiologic alterations of respiratory, cardiovascular, neurological, metabolic, musculoskeletal, hepatic and renal functions [11, 12], which in part may be reflected in various circulating blood biomarkers [13–15]. In context of a large-scale geroscience-guided clinical trial that aims to examine the effects of metformin treatment on the incidence of age-related chronic diseases and functional degeneration [16], an expert panel performed a comprehensive literature review and identified a shortlist of about 10 selected blood-based biomarkers that may be used to monitor ageing processes [15]. The 10 markers were selected from initially 258 candidates, based on criteria of (1) measurement reliability and feasibility, (2) relevance to ageing, (3) ability to predict all-cause mortality, clinical and functional outcomes, and (4) potential responsiveness to lifestyle or medical interventions. Five major biomarkers in the selected list are: (i) growth differentiation factor-15 (GDF-15; previously also known as macrophage-inhibitory cytokine-1 [MIC-1]), produced in response to oxidative stress and mitochondrial dysfunction and used as a marker for metabolic effects induced by metformin, a widely used glucose-lowering agent with a potential life-span extending effects in animal studies [17, 18]; (ii) N-terminal pro-brain natriuretic peptide (NT-proBNP), a protein secreted by ventricular myocytes to decrease vascular resistance [19] and used as biomarker for cardiovascular health, to diagnose and establish the prognosis for heart failure [20]; (iii) glycated hemoglobin A1c (HbA1C), a marker for medium-term average plasma glucose and metabolic ageing [15]; (iv) C-reactive protein (CRP), a marker for systemic inflammation [15]; and (v) cystatin-C, a biomarker of glomerular filtration rate (GFR), kidney disease and ageing-related physical and cognitive dysfunction [21].

For these 5 selected biomarkers, examined either individually or in various combinations [22, 23], epidemiologic studies have documented clear associations with increased CVD risk and mortality, summarized in several meta-analyses [24–28]. With regard to cancer risk, studies have also documented associations of higher CRP concentrations with increased risks of cancers of the lung [29, 30], breast [31] and colorectum [32], but not prostate cancer [33], and of elevated HbA1C levels with increased risks of endometrial, renal, colorectal, gastric, pancreatic, breast, liver and respiratory cancers [34–36]. By contrast, only few studies so far have examined the possible relationships of cancer risk with GDF-15 [37–41], NT-proBNP [42], and Cystatin-C levels [43], and with inconsistent results. In light of the limited investigations on how these ageing-related biomarkers, alone or in combination may be associated with cancer risk, we conducted a prospective case-cohort study within the European Prospective Investigation into Cancer and Nutrition (EPIC)–Heidelberg cohort to assess the long-term associations between blood levels of GDF-15, NT-proBNP, HbA1C, CRP and Cystatin-C with the risk of the four most frequent forms of cancer (breast, prostate, colorectum, lung). For comparison, we performed parallel analyses on these markers in relation the risks of myocardial infarction (MI) and stroke. The aim of these exploratory analyses is to examine whether these markers related to different dimensions of ageing (such as inflammation, mitochondrial dysfunction, metabolic and functional ageing), and having shown strong associations with chronological age and mortality risk, are associated with the risks of cancer and CVD.

## Methods

### Study setting

The current study used a nested case-cohort design, embedded within the EPIC-Heidelberg Study– a population-based cohort study that was initiated to investigate associations between diet, metabolic factors and lifestyle with the risks of cancer and other chronic diseases [44]. The EPIC-Heidelberg cohort comprises a total of 25,540 women and men aged 35–65 recruited between 1994 and 1998 from the local general population in Heidelberg and surrounding municipalities. At baseline, data about participants' health status, usual diet, lifestyle, socioeconomic status, and reproductive history were collected using extensive self-administered questionnaires and interviews, and anthropometric indices (height, weight, waist and hip circumferences) were measured. A blood sample was drawn on the day of recruitment, independently of fasting status, and kept for a maximum of 24 h at + 4 °C to + 10 °C until centrifugation and further processing. Blood samples were aliquoted into fractions of plasma, serum, erythrocytes and buffy coat and stored under liquid nitrogen at − 196 °C. Informed consent was obtained from all participants at baseline.

### Prospective disease ascertainment

In EPIC–Heidelberg, incident chronic disease occurrences were prospectively ascertained through active follow-up among study subjects and their next-of-kin, combined with linkages to hospitalization records, and cancer and pathology registries. Mortality outcomes were ascertained from death certificates which were collected from mortality registries. For the present case-cohort study, all verified incident cases of breast (International Classification of Diseases (ICD)-10: C50, 685 cases), prostate (ICD-10: C61, 597 cases), lung (ICD-10: C34, 219 cases) and colorectal (ICD-10: C18-20, 284 cases) cancer, as well as incident cases of MI (ICD-10: I21, 774 cases) and stroke (ICD-10: I60, I61, I63, I64, 798 cases), diagnosed up to the end of December 2014 were included. All cases were validated and coded by a study physician based on medical records and only verified cases remained in the dataset. In addition, to reach exhaustiveness, all cases detected via the linkage to cancer registries were included, even if they were not self-reported. For breast cancer, we also abstracted clinical record data about tumor estrogen receptor (ER), progesterone receptor (PR) and human epidermal growth factor receptor (Her2) status. For prostate cancer we used clinical record data to further sub-classify into high- and low-grade disease based on Gleason scores (high grade: Gleason Score = 4 + 3 or ≥ 8, low grade: Gleason Score = 3 + 4 or ≤ 6) [45].

### Case-cohort sampling

The sub-cohort was selected using a two-step age-stratified sampling of the entire EPIC-Heidelberg cohort (for details, see Supplemental Material, Figure S1). Case-cohort designs allow investigating several different outcomes, and sparing the excessive use of biological specimens [46]. Oversampling older participants ensures a stronger statistical power to investigate age-related outcomes. The first sampling step (2009 case-cohort) consisted of a 10% random selection (Sub1) of the full cohort, which was used for initial case-cohort analyses with chronic disease cases diagnosed until December 2009 [47, 48]. For the second step (2014 case-cohort), an additional 10% sampling (Sub2) was performed among participants who at baseline were above age 50 and who had not been sampled during the first step. The 2 samples (Sub1 and Sub2) were then merged to obtain the final sub-cohort, with a total of 3794 randomly selected study participants. The present case-cohort study further included a total of 4869 verified cases of cancer (breast, colorectum, prostate, lung) or CVD (myocardial infarction, stroke) occurring until the end of December 2014. Of the incident disease cases, 894 occurred within the sub-cohort; thus, the present case-cohort study is based on a total of 7783 study participants, among which 7767 had data for at least one of the biomarkers of interest (2 participants were excluded from the sub-cohort and 16 from the cases because they had no biomarker measurement available).

### Laboratory measurements

The measurements of GDF-15, NT-proBNP, CRP and cystatin-C were carried out on the electrochemiluminescence platform QuickPlex SQ 120 (Meso Scale Diagnostics (MSD), Maryland, USA). R-Plex kits for each of the analytes were obtained from MSD and the protocols carried out according to the manufacturer’s primary protocol. Briefly, the biotin coupled capture antibody was incubated in small spot streptavidin coated plates followed by wash steps and incubation of the calibrators, quality controls (QCs) and study samples at appropriate dilutions (GDF-15 1:100, NT-proBNP 1:20, CRP 1:1000 and Cystatin-C 1:1000). After three wash steps, the wells were incubated with a secondary detection antibody conjugated to the electrochemiluminescent label. Following three further washes, the plates were incubated with MSD Gold read buffer and analyzed in the QuickPlex SQ 120 instrument. The coefficient of variation (CV) of QCs for inter-batch and intra-batch measurements were 2,0% and 12.8% for GDF-15, 3.9% and 18.8% for NT-proBNP, 4.0% and 15.9% for CRP and 6.5% and 20.3% for Cystatin C. HbA1C samples were analyzed on the HPLC Variant Turbo II (Bio-Rad, Munich Germany) according to the manufacturer’s instructions. After installation of a fresh analytical cartridge, the system was primed with the provided whole blood samples and calibrated with provided standards. Unknown samples were then run batch wise using an auto-sampler with QCs inserted throughout each batch. The CV percentage for HbA1c QCs was 2.01% inter-batch while intra-batch CV was 3.89%. Laboratory personnel were blinded to sample type/status for all measurements and assays.

### Statistical analyses

Participants’ characteristics at baseline were described separately for cohort study participants selected in the sub-cohort (according to sex), and for the cases of each of the selected chronic disease endpoints. Spearman correlation coefficients, and linear regression analyses were used to examine associations of GDF-15, NT-proBNP, HbA1C, CRP and cystatin-C with age, sex, level of education, physical activity level, body mass index (BMI), smoking (status, lifetime duration, pack-years), alcohol consumption, and baseline type-2 diabetes, and to estimate the percentage of the variance (adjusted model R2) in each biomarker potentially explained by lifestyle factors.

Prospective associations between the five biomarkers and risks of breast, prostate, lung, colorectal cancers, MI and stroke, were assessed using Cox proportional hazards models. Inverse sub-cohort sampling probability (ISSP) [46] weighting was used to account for the case-cohort design, and for the oversampling of older participants according to the case-cohort sampling scheme; that is, participants aged 50 or younger were assigned a 10% probability, and those aged above 50 a 19% probability (10% given they were not drawn in the first selection step (a 90% probability): 10% + (10% × 90%)). Cause-specific hazard ratios (HR) and their 95% confidence intervals (CI) were obtained for any first occurrence of incident cancer (i.e., considering the earlier occurrence of any other cancer type as competing events, with the exception of non-melanoma skin cancer) or incident cardiovascular event (where stroke and MI were considered as mutually competing events). Cancers and CVD were not considered as mutually competing events. In all Cox models, age was the underlying timescale, and all models were additionally stratified by age at recruitment (5-year category) to account for a potential birth cohort effect. Biomarkers were used both as categorical (sex-specific quartiles based on the distribution in the sub-cohort), and continuous (after a log-2 based transformation; HRs were therefore interpreted as the relative hazard for a doubling of biomarker concentration). The proportional hazard assumption was tested using an extended version of the Schoenfeld residuals [49], and tests for linear trends were based on the median of each quartile modelled as a continuous variable.

Model 1 (intrinsically adjusted for age as timescale) was adjusted for sex (except for breast and prostate cancer) and age-stratified. To account for possible confounding factors, a further model (model 2) was additionally adjusted for BMI (kg/m^2^, continuous), lifetime alcohol consumption (g/day, continuous), smoking status (never, long time quitters, short time quitters, current light, and current heavy smokers), physical activity (inactive, moderately inactive, moderately active, active), educational level (none/primary school, technical school, secondary school, university), baseline self-reported diabetes (yes/no) (only for GDF-15 and HbA1C), and baseline self-reported hypertension (yes/no) (only for MI and stroke analyses). All confounders were completely known with no missing data. Finally, we fitted mutually adjusted models in which, for each disease outcome, all 5 biomarkers were included as continuous variables (log-2 transformed) to explore which markers showed associations with each disease after adjusting for the other biomarkers. The latter models were then used to build models with multi-marker combinations, with significant markers weighted by their relative beta-coefficients. We explored the magnitude of the associations between quartiles of this combination index and risks of cancer and CVD.

In secondary analyses, we investigated the associations between the biomarker levels and risks of breast cancer according to tumor subtypes (ER-, Her2 + , luminal [ER + /Her2-] and triple negative [ER-/PR-/Her2-] tumors) and age at diagnosis (using a cut-off of 55, as a proxy for menopausal status), and prostate cancer according to the grade of tumor (low and high as described earlier). In addition, associations were explored separately by smoking status (never, former, current). To assess possible reverse causation bias, sensitivity analyses were conducted excluding cases diagnosed within the first 2 years of follow-up. Tests were two-sided and p-values less than 0.05 were considered statistically significant. All analyses were performed using SAS V.9.4 (SAS Institute).

## Results

Baseline characteristics of the EPIC–Heidelberg sub-cohort participants, having data for at least one of the five biomarkers (n = 3792) are described in Table 1. For women (51%) in the sub-cohort, the average age at enrollment was 51 years (range 35–66), whereas for men the average age was 54 years (range 40–65). Both women and men on average were slightly overweight, whereas among women, almost two-thirds of the participants reported to be at least moderately active, and 37% among men. Among women, 53% had never smoked and 22% had a university degree, whereas among men, 31% had never smoked and 36% had a university degree. The prevalence rates of self-reported type-2 diabetes and hypertension were 2 and 27%, respectively, among women, and 6 and 37%, respectively, among men.

**Table 1:** Baseline characteristics of the study population, EPIC-Heidelberg Case-cohort (n = 7767)

	Subcohort			Incident cases					
	Total	Men	Women	Breast cancer	Prostate cancer	Lung cancer	Colorectal cancer	MI	Stroke
	(n = 3792)	(n = 1847)	(n = 1945)	(n = 684)	(n = 596)	(n = 218)	(n = 283)	(n = 740)	(n = 758)
Cases in subcohort	n = 894	n = 563	n = 331	n = 110	n = 116	n = 40	n = 68	n = 128	n = 139
Age at recruitment*	53 (35; 66)	54 (40; 65)	51 (35; 66)	51 (35; 65)	57 (41; 65)	54 (36; 65)	55 (36; 65)	55 (36; 66)	56 (35; 65)
Duration of follow-up	17 ± 3	17 ± 4	17 ± 2	9 ± 5	9 ± 5	10 ± 5	9 ± 4	10 ± 5	10 ± 5
Smoking status									
Never	1599 (42%)	573 (31%)	1026 (53%)	345 (50%)	232 (39%)	17 (8%)	95 (34%)	218 (29%)	265 (35%)
Long time quitter (> 10 y ago)	1037 (27%)	663 (36%)	374 (19%)	141 (21%)	216 (36%)	33 (15%)	88 (31%)	189 (25%)	218 (29%)
Short time quitter (≤ 10 y or less)	353 (9%)	202 (11%)	151 (8%)	64 (9%)	56 (9%)	23 (11%)	35 (12%)	74 (10%)	65 (9%)
Current, light (≤ 10 cig /d)	277 (7%)	104 (6%)	173 (9%)	59 (9%)	26 (4%)	24 (11%)	26 (9%)	64 (9%)	59 (8%)
Current, heavy (> 10 cig/d)	526 (14%)	305 (17%)	221 (11%)	75 (11%)	66 (11%)	121 (55%)	39 (14%)	195 (26%)	151 (20%)
Level of education									
None/primary school completed	1156 (31%)	585 (32%)	571 (29%)	174 (25%)	205 (34%)	101 (46%)	102 (36%)	289 (39%)	289 (38%)
Technical/professional school	1302 (34%)	500 (27%)	802 (41%)	272 (40%)	174 (29%)	77 (35%)	91 (32%)	229 (31%)	246 (32%)
Secondary school	229 (6%)	94 (5%)	135 (7%)	57 (8%)	16 (3%)	11 (5%)	14 (5%)	29 (4%)	34 (4%)
Longer university (incl. University)	1105 (29%)	668 (36%)	437 (22%)	181 (26%)	201 (34%)	29 (13%)	76 (27%)	193 (26%)	189 (25%)
Physical activity level									
Inactive	838 (22%)	594 (32%)	244 (12%)	97 (14%)	182 (30%)	35 (16%)	69 (24%)	176 (24%)	159 (21%)
Moderately inactive	1078 (28%)	582 (32%)	496 (25%)	190 (28%)	183 (31%)	78 (36%)	66 (23%)	215 (29%)	215 (28%)
Moderately active	1569 (41%)	557 (31%)	1012 (52%)	334 (49%)	203 (34%)	88 (40%)	120 (42%)	285 (38%)	318 (42%)
Active	307 (8%)	114 (6%)	193 (10%)	63 (9%)	28 (5%)	17 (8%)	28 (10%)	64 (9%)	66 (9%)
Baseline self-reported diabetes									
Yes	152 (4%)	109 (6%)	43 (2%)	9 (1%)	30 (5%)	9 (4%)	22 (8%)	80 (11%)	65 (9%)
Baseline self-reported hypertension									
Yes	1205 (32%)	683 (37%)	522 (27%)	172 (25%)	219 (37%)	75 (34%)	111 (39%)	328 (44%)	352 (46%)
BMI (Kg/m^2^)	26.2 ± 4.3	26.9 ± 3.6	25.5 ± 4.7	25.3 ± 4.4	27.0 ± 3.2	26.5 ± 4.4	27.4 ± 3.9	28.0 ± 4.0	27.2 ± 4.3
Alcohol consumption (g/d)	17 ± 27	28 ± 32	7 ± 15	8 ± 10	27 ± 27	36 ± 51	25 ± 31	22 ± 27	22 ± 27
GDF-15 (pg/mL)	730 ± 1143	727 ± 487	733 ± 1527	702 ± 1421	719 ± 436	931 ± 487	715 ± 335	799 ± 426	830 ± 534
NT-proBNP (pg/mL)	235 ± 323	204 ± 370	263 ± 269	298 ± 273	233 ± 331	220 ± 251	262 ± 322	262 ± 367	295 ± 398
HbA1C (mmol/mol)^∞^	36 ± 8	37 ± 9	35 ± 7	35 ± 5	37 ± 9	38 ± 8	37 ± 9	39 ± 12	38 ± 9
CRP (mg/L)	3 ± 5	3 ± 5	3 ± 5	3 ± 4	3 ± 5	5 ± 5	4 ± 6	5 ± 6	4 ± 5
Cystatin-C (ng/mL)	511 ± 356	532 ± 414	491 ± 291	467 ± 402	564 ± 317	533 ± 289	538 ± 301	570 ± 366	561 ± 308

Values are n (%) for categorical variables, and mean ± SD for continuous variables

^*^mean (min; max)

^∞^HbA1C in % can be obtained from mmol/mol as follows: HbA1C (%) = (0.0915 × (HbA1C mmol/mol)) + 2.15%

In the sub-cohort, all five biomarkers showed significant associations with age (Table 2); however, the association was not linear for NT-proBNP. Age and sex in combination explained respectively 13, 4, 7, 2 and 13% percent of the variance of GDF-15, NT-proBNP, HbA1C, CRP and cystatin-C. Among the other covariates, the strongest predictors of the five biomarkers were smoking status for GDF-15 (explaining 8% of the variance of GDF-15, after adjustment for age and sex), and baseline diabetes for HbA1C (explaining 28% after adjustment for age and sex). When analyzed simultaneously, all the covariates explained respectively 23, 4, 37, 12 and 14% of the variance in circulating GDF-15, NT-proBNP, HbA1C, CRP and cystatin-C. The biomarkers were not strongly correlated with each other: strongest correlations were found between GDF-15 and CRP (age and sex-adjusted Spearman partial correlation coefficient = 0.26) and HbA1C and CRP (coefficient = 0.19) (Supplemental Figure S2).

**Table 2:** Levels of GDF-15, NT-proBNP, HbA1C, CRP, cystatin-C among different strata of the sub-cohort, EPIC-Heidelberg (n = 3792)

	GDF-15			NT-proBNP			HbA1C			CRP			Cystatin-C		
	Mean ± SE	P*	R^2^	Mean ± SE	P*	R^2^	Mean ± SE	P*	R^2^	Mean ± SE	P*	R^2^	Mean ± SE	P*	R^2^
*Age*		< .001	0.13		< .001	0.04		< .001	0.07		< .001	0.02		< .001	0.13
< 40	518 ± 18			212 ± 21			33.3 ± 0.5			2.1 ± 0.3			337 ± 18		
[40—45[	533 ± 12			205 ± 13			33.9 ± 0.3			2.6 ± 0.2			350 ± 11		
[45—50[	586 ± 13			218 ± 14			34.5 ± 0.3			2.2 ± 0.2			356 ± 12		
[50—55[	645 ± 9			180 ± 11			35.8 ± 0.3			3.1 ± 0.2			563 ± 9		
[55 -60[	731 ± 9			224 ± 11			36.7 ± 0.3			3.7 ± 0.2			589 ± 9		
≥ 60	803 ± 10			315 ± 11			38.3 ± 0.3			4.0 ± 0.2			597 ± 10		
*Sex*		< .001	0.13		< .001	0.04		< .001	0.07		0.07	0.02		0.94	0.13
Men	661 ± 7			196 ± 8			35.9 ± 0.2			2.8 ± 0.1			465 ± 7		
Women	611 ± 6			255 ± 7			34.9 ± 0.2			3.1 ± 0.1			465 ± 6		
*Level of education*		< .001	0.14		< .001	0.04		< .001	0.08		< .001	0.03		0.80	0.13
None/primary school	676 ± 8			225 ± 10			36.5 ± 0.2			3.6 ± 0.1			470 ± 8		
Technical/professional	631 ± 8			243 ± 9			35.8 ± 0.2			2.9 ± 0.1			459 ± 8		
Secondary school	655 ± 18			222 ± 21			35.1 ± 0.5			2.9 ± 0.3			467 ± 17		
Longer (incl. University)	601 ± 8			210 ± 9			34.2 ± 0.2			2.4 ± 0.1			465 ± 8		
*Physical activity level*		0.60	0.13		0.64	0.04		0.05	0.07		0.45	0.02		0.53	0.13
Inactive	630 ± 10			218 ± 12			34.8 ± 0.5			2.7 ± 0.2			477 ± 9		
Moderately inactive	640 ± 9			231 ± 10			35.5 ± 0.2			3.1 ± 0.2			464 ± 8		
Moderately active	640 ± 7			230 ± 9			35 .7 ± 0.2			3.0 ± 0.1			461 ± 7		
Active	620 ± 15			210 ± 18			35.5 ± 0.4			3.1 ± 0.3			454 ± 15		
*BMI*		< .001	0.13		0.31	0.04		< .001	0.09		< .001	0.09		0.53	0.13
Normal	642 ± 7			225 ± 8			34.7 ± 0.2			2.0 ± 0.1			464 ± 7		
Overweight	614 ± 8			219 ± 9			35.2 ± 0.2			3.3 ± 0.1			463 ± 7		
Obese	674 ± 11			243 ± 14			38.1 ± 0.3			5.1 ± 0.2			477 ± 11		
*Smoking status*		< .001	0.21		0.67	0.04		< .001	0.08		< .001	0.04		0.01	0.13
Never	579 ± 7			218 ± 8			34.8 ± 0.2			2.6 ± 0.1			459 ± 7		
Long time quitter (> 10 y)	598 ± 9			229 ± 11			35.0 ± 0.2			2.7 ± 0.2			450 ± 9		
Short time quitter (≤ 10 y)	643 ± 14			247 ± 17			35.4 ± 0.4			3.2 ± 0.3			469 ± 14		
Current, light (≤ 10 cig /d)	679 ± 16			225 ± 19			36.2 ± 0.5			2.6 ± 0.3			470 ± 16		
Current, heavy (> 10 cig/d)	824 ± 11			225 ± 14			37.3 ± 0.3			4.3 ± 0.2			501 ± 12		
*Alcohol consumption*^£^		0.008	0.14		0.32	0.04		< .001	0.07		0.42	0.02		0.72	0.13
Low consumers	624 ± 7			220 ± 7			35.9 ± 0.2			2.9 ± 0.1			467 ± 7		
High consumers	647 ± 7			231 ± 7			35.0 ± 0.2			3.0 ± 0.1			464 ± 6		
*Baseline diabetes*		< .001	0.15		0.99	0.04		< .001	0.35		< .001	0.02		0.27	0.13
No	629 ± 5			226 ± 5			34.6 ± 0.1			2.9 ± 0.1			466 ± 5		
Yes	841 ± 23			226 ± 28			59.6 ± 0.5			4.7 ± 0.4			441 ± 23		

GDF-15: n = 3701; NT-proBNP: n = 3430; HbA1C: n = 3717; CRP: n = 3635; Cystatin-C: n = 3665

HbA1C in % can be obtained from mmol/mol as follows: HbA1C (%) = (0.0915 × (HbA1C mmol/mol)) + 2.15%

Values are means (adjusted for age and sex) ± SE

^*^p-value for lifestyle factor, obtained using a generalized linear model adjusted for age and sex

R^2^: adjusted R^2^ obtained using age and sex-adjusted linear regressions, interpreted as the variance in the biomarker jointly explained by age, sex and the specific lifestyle factor

^£^Lifetime alcohol consumption according to sex-specific median: median in men = 20.6 g/day and in women = 4.1 g/day

R^2^ for a model including all of the above factors: 0.23 for GDF-15, 0.04 for NT-proBNP, 0.37 for HbA1C, 0.12 for CRP and 0.14 for Cystatin-C

Regarding CVD, proportional hazards models adjusted only for age and sex showed significant increases in risks of both MI and stroke at higher levels of each of the five biomarkers. Highest hazard ratios were observed for CRP, HbA1c and GDF-15, with hazard ratios above 2.4 for MI, and above 1.6 for stroke, when comparing highest to lowest quartile levels of these markers (Table 3). Additional adjustments for BMI, lifetime alcohol consumption, smoking (never, long time quitters, short time quitters, current light, and current heavy smokers), physical activity level, educational level, baseline self-reported diabetes (for GDF-15 and HbA1C), and baseline self-reported hypertension (model 2) only moderately attenuated the HR estimates for each of the markers, and after full multivariable adjustment only the association of HbA1C with risk of stroke was no longer statistically significant.

**Table 3:** Associations between GDF-15, NT-proBNP, HBA1C, CRP, cystatin-C with cancer and CVD risk, EPIC-Heidelberg (n = 7767)

	Q1	Q2	Q3	Q4	P-trend	Continuous	P
GDF-15							
*Breast cancer*							
Number of cases	145	175	156	147			
Model 1	Ref	1.20 (0.92,1.55)	1.11 (0.84,1.46)	1.11 (0.84,1.47)	0.72	1.00 (0.88,1.14)	0.98
Model 2	Ref	1.22 (0.94,1.59)	1.15 (0.87,1.53)	1.19 (0.89,1.61)	0.42	1.03 (0.90,1.17)	0.71
*Prostate cancer*							
Number of cases	134	140	167	136			
Model 1	Ref	0.80 (0.60,1.05)	0.80 (0.61,1.05)	0.66 (0.50,0.89)	0.01	0.76 (0.62,0.94)	0.01
Model 2	Ref	0.83 (0.63,1.10)	0.84 (0.63,1.11)	0.71 (0.52,0.97)	0.05	0.79 (0.63,0.99)	0.04
*Lung cancer*							
Number of cases	19	28	45	110			
Model 1	Ref	1.66 (0.91,3.03)	2.85 (1.60,5.09)	8.14 (4.70,14.09)	< .001	2.10 (1.77,2.50)	< .001
Model 2	Ref	1.10 (0.59,2.03)	1.27 (0.70,2.31)	2.73 (1.57,4.77)	< .001	1.64 (1.37,1.96)	< .001
*Colorectal cancer*							
Number of cases	45	76	73	74			
Model 1	Ref	1.45 (0.98,2.14)	1.24 (0.83,1.86)	1.29 (0.85,1.95)	0.56	1.02 (0.82,1.28)	0.86
Model 2	Ref	1.34 (0.90,2.00)	1.09 (0.72,1.66)	1.06 (0.68,1.66)	0.73	0.91 (0.71,1.16)	0.45
*Mycordial Infarction*							
Number of cases	106	142	224	236			
Model 1	Ref	1.30 (0.98,1.72)	2.00 (1.53,2.61)	2.39 (1.82,3.14)	< .001	1.66 (1.46,1.87)	< .001
Model 2	Ref	1.10 (0.83,1.46)	1.47 (1.11,1.94)	1.43 (1.07,1.91)	0.03	1.32 (1.14,1.54)	< .001
*Stroke*							
Number of cases	102	141	193	288			
Model 1	Ref	1.24 (0.94,1.65)	1.56 (1.18,2.05)	2.51 (1.91,3.29)	< .001	1.65 (1.46,1.86)	< .001
Model 2	Ref	1.15 (0.87,1.53)	1.33 (1.01,1.77)	1.93 (1.44,2.57)	< .001	1.47 (1.28,1.68)	< .001
NT-proBNP							
*Breast cancer*							
Number of cases	103	161	159	179			
Model 1	Ref	1.61 (1.21,2.14)	1.61 (1.21,2.15)	1.80 (1.37,2.38)	< .001	1.18 (1.11,1.27)	< .001
Model 2	Ref	1.63 (1.23,2.17)	1.63 (1.21,2.18)	1.83 (1.38,2.44)	< .001	1.19 (1.11,1.27)	< .001
*Prostate cancer*							
Number of cases	89	126	157	177			
Model 1	Ref	1.49 (1.10,2.01)	1.91 (1.42,2.57)	1.92 (1.44,2.57)	< .001	1.17 (1.10,1.25)	< .001
Model 2	Ref	1.48 (1.09,2.02)	1.90 (1.40,2.56)	1.90 (1.42,2.56)	< .001	1.17 (1.10,1.25)	< .001
*Lung cancer*							
Number of cases	40	50	54	47			
Model 1	Ref	1.25 (0.82,1.92)	1.33 (0.87,2.03)	1.21 (0.78,1.87)	0.78	1.08 (0.97,1.19)	0.15
Model 2	Ref	1.24 (0.79,1.95)	1.39 (0.89,2.19)	1.12 (0.71,1.76)	0.97	1.06 (0.96,1.18)	0.23
*Colorectal cancer*							
Number of cases	42	68	57	90			
Model 1	Ref	1.76 (1.17,2.63)	1.51 (0.99,2.30)	2.21 (1.50,3.25)	< .001	1.19 (1.10,1.30)	< .001
Model 2	Ref	1.76 (1.17,2.65)	1.52 (1.00,2.32)	2.22 (1.50,3.28)	< .001	1.19 (1.10,1.30)	< .001
*Mycordial Infarction*							
Number of cases	136	154	175	207			
Model 1	Ref	1.15 (0.89,1.48)	1.31 (1.02,1.69)	1.53 (1.20,1.96)	< .001	1.15 (1.08,1.23)	< .001
Model 2	Ref	1.18 (0.90,1.54)	1.35 (1.04,1.76)	1.62 (1.26,2.08)	< .001	1.16 (1.09,1.24)	< .001
*Stroke*							
Number of cases	144	147	176	227			
Model 1	Ref	1.06 (0.83,1.37)	1.26 (0.98,1.61)	1.51 (1.19,1.91)	< .001	1.16 (1.09,1.24)	< .001
Model 2	Ref	1.07 (0.82,1.38)	1.24 (0.96,1.60)	1.48 (1.17,1.88)	< .001	1.15 (1.08,1.23)	< .001
HbA1C							
*Breast cancer*							
Number of cases	178	147	189	161			
Model 1	Ref	1.26 (0.98,1.63)	1.29 (1.01,1.65)	1.30 (1.01,1.67)	0.06	1.24 (0.87,1.76)	0.24
Model 2	Ref	1.29 (1.00,1.66)	1.34 (1.04,1.72)	1.43 (1.09,1.88)	0.01	1.78 (1.08,2.94)	0.02
*Prostate cancer*							
Number of cases	179	104	149	153			
Model 1	Ref	0.99 (0.75,1.31)	1.17 (0.91,1.51)	1.06 (0.82,1.37)	0.48	1.00 (0.72,1.38)	0.99
Model 2	Ref	1.02 (0.77,1.35)	1.19 (0.92,1.54)	1.21 (0.92,1.60)	0.12	1.35 (0.90,2.03)	0.15
*Lung cancer*							
Number of cases	37	31	69	76			
Model 1	Ref	1.45 (0.89,2.37)	2.83 (1.86,4.32)	3.28 (2.13,5.07)	< .001	2.58 (1.84,3.63)	< .001
Model 2	Ref	1.16 (0.70,1.93)	1.92 (1.21,3.04)	1.74 (1.07,2.81)	0.02	2.66 (1.32,5.34)	0.01
*Colorectal cancer*							
Number of cases	77	46	73	83			
Model 1	Ref	0.97 (0.66,1.41)	1.19 (0.85,1.66)	1.36 (0.97,1.90)	0.05	1.62 (1.07,2.46)	0.02
Model 2	Ref	0.93 (0.64,1.37)	1.14 (0.80,1.61)	1.09 (0.74,1.61)	0.50	1.17 (0.67,2.05)	0.58
*Mycordial Infarction*							
Number of cases	164	113	186	265			
Model 1	Ref	1.17 (0.90,1.52)	1.63 (1.29,2.06)	2.42 (1.93,3.04)	< .001	3.02 (2.31,3.95)	< .001
Model 2	Ref	1.06 (0.81,1.38)	1.39 (1.09,1.77)	1.50 (1.16,1.93)	< .001	1.92 (1.29,2.88)	< .001
*Stroke*							
Number of cases	177	128	187	221			
Model 1	Ref	1.17 (0.91,1.49)	1.37 (1.09,1.72)	1.59 (1.26,2.00)	< .001	1.95 (1.48,2.57)	< .001
Model 2	Ref	1.10 (0.86,1.42)	1.25 (0.99,1.58)	1.18 (0.91,1.52)	0.22	1.38 (0.92,2.07)	0.12
CRP							
*Breast cancer*							
Number of cases	151	161	157	148			
Model 1	Ref	1.09 (0.85,1.41)	1.07 (0.83,1.38)	1.04 (0.80,1.35)	0.96	1.00 (0.95,1.05)	0.86
Model 2	Ref	1.16 (0.89,1.50)	1.18 (0.90,1.55)	1.17 (0.88,1.56)	0.54	1.02 (0.96,1.08)	0.50
*Prostate cancer*							
Number of cases	130	137	163	142			
Model 1	Ref	0.98 (0.74,1.29)	1.11 (0.85,1.44)	0.97 (0.74,1.28)	0.82	1.02 (0.96,1.08)	0.52
Model 2	Ref	1.04 (0.78,1.39)	1.18 (0.89,1.56)	1.09 (0.80,1.48)	0.71	1.05 (0.98,1.11)	0.16
*Lung cancer*							
Number of cases	23	37	52	85			
Model 1	Ref	1.68 (0.98,2.87)	2.37 (1.43,3.93)	4.15 (2.55,6.75)	< .001	1.32 (1.22,1.43)	< .001
Model 2	Ref	1.37 (0.78,2.40)	1.67 (0.97,2.86)	2.25 (1.31,3.87)	< .001	1.16 (1.05,1.28)	< .001
*Colorectal cancer*							
Number of cases	46	58	78	77			
Model 1	Ref	1.19 (0.79,1.77)	1.51 (1.02,2.22)	1.52 (1.03,2.25)	0.05	1.10 (1.02,1.19)	0.01
Model 2	Ref	1.09 (0.72,1.65)	1.33 (0.89,1.99)	1.26 (0.81,1.96)	0.40	1.06 (0.97,1.16)	0.18
*Mycordial Infarction*							
Number of cases	93	125	192	286			
Model 1	Ref	1.35 (1.01,1.80)	2.07 (1.57,2.72)	3.34 (2.57,4.34)	< .001	1.30 (1.24,1.37)	< .001
Model 2	Ref	1.15 (0.86,1.55)	1.59 (1.19,2.11)	2.15 (1.61,2.87)	< .001	1.19 (1.12,1.25)	< .001
*Stroke*							
Number of cases	105	140	204	263			
Model 1	Ref	1.29 (0.98,1.69)	1.78 (1.37,2.31)	2.41 (1.87,3.11)	< .001	1.19 (1.14,1.25)	< .001
Model 2	Ref	1.19 (0.90,1.57)	1.56 (1.20,2.04)	1.91 (1.45,2.52)	< .001	1.14 (1.08,1.20)	< .001
Cystatin-C							
*Breast cancer*							
Number of cases	172	162	141	143			
Model 1	Ref	0.94 (0.72,1.22)	0.81 (0.61,1.07)	0.85 (0.63,1.15)	0.29	0.86 (0.74,0.99)	0.04
Model 2	Ref	0.95 (0.73,1.24)	0.82 (0.61,1.09)	0.87 (0.64,1.18)	0.35	0.86 (0.75,1.00)	0.05
*Prostate cancer*							
Number of cases	111	159	130	164			
Model 1	Ref	1.18 (0.89,1.56)	0.86 (0.64,1.15)	1.05 (0.79,1.40)	0.86	1.02 (0.90,1.17)	0.72
Model 2	Ref	1.18 (0.89,1.56)	0.86 (0.64,1.15)	1.06 (0.79,1.41)	0.89	1.03 (0.90,1.17)	0.72
*Lung cancer*							
Number of cases	43	46	59	50			
Model 1	Ref	1.08 (0.68,1.69)	1.39 (0.90,2.14)	1.18 (0.74,1.86)	0.45	1.13 (0.93,1.38)	0.20
Model 2	Ref	1.03 (0.64,1.67)	1.22 (0.77,1.94)	1.06 (0.65,1.72)	0.80	1.08 (0.88,1.33)	0.47
*Colorectal cancer*							
Number of cases	52	70	70	72			
Model 1	Ref	1.18 (0.81,1.71)	1.09 (0.74,1.60)	1.06 (0.72,1.56)	0.96	0.99 (0.83,1.18)	0.93
Model 2	Ref	1.15 (0.79,1.68)	1.08 (0.73,1.59)	1.03 (0.70,1.51)	0.84	0.99 (0.83,1.18)	0.90
*Mycordial Infarction*							
Number of cases	145	172	166	212			
Model 1	Ref	1.17 (0.91,1.50)	1.13 (0.87,1.45)	1.40 (1.09,1.81)	0.01	1.23 (1.09,1.39)	< .001
Model 2	Ref	1.13 (0.87,1.46)	1.04 (0.80,1.35)	1.25 (0.97,1.61)	0.12	1.17 (1.04,1.32)	0.01
*Stroke*							
Number of cases	130	182	201	203			
Model 1	Ref	1.26 (0.98,1.61)	1.34 (1.05,1.72)	1.28 (0.99,1.66)	0.16	1.16 (1.04,1.30)	0.01
Model 2	Ref	1.21 (0.94,1.56)	1.27 (0.99,1.64)	1.21 (0.94,1.57)	0.32	1.14 (1.01,1.28)	0.03

Quartiles are based on the sex-specific distribution of the biomarker in the sub-cohort

Sex-specific quartile cut-offs for GDF-15 were 503.1, 628.6, 830.0 in men and 451.2, 576.6, 737.4 in women

Sex-specific quartile cut-offs for NT-proBNP were 61.3, 114.7, 230.8 in men and 93.3, 193.4, 338.0 in women

Sex-specific quartile cut-offs for HbA1C were 33, 35, 38 in men and 32, 34, 37 in women

Sex-specific quartile cut-offs for CRP were 0.7, 1.6, 3.5 in men and 0.6, 1.5, 3.6 in women

Sex-specific quartile cut-offs for Cystatin-C were 324.7, 458.2, 647.8 in men and 294.2, 418.1, 603.7 in women

Model 1 is a cause-specific Cox model adjusted for sex (except for breast and prostate cancer) and age (as timescale), and stratified for age as 5-y categories

Model 2 is further adjusted for BMI, lifetime alcohol consumption, smoking status (never, long time quitters, short time quitters, current light, and current heavy smokers), physical activity level, educational level, baseline self-reported diabetes (for GDF-15 and HbA1C), and baseline self-reported hypertension (for myocardial infarction and stroke)

Continuous HR for one unit increment in log-2 based biomarker = change in hazard associated with a doubling of biomarker concentration

HRs were corrected to match case-cohort design using inverse sub-cohort sampling probability weighting (ISSP)

With regard to cancer, basic risk models adjusted only for age and sex showed increased risks at multiple cancer (organ) sites for higher blood concentrations of NT-proBNP (breast, prostate, colorectum), HbA1C (lung, colorectum, breast), and CRP (lung, colorectum), whereas higher GDF-15 levels were associated only with an increased risk of lung cancer. The strongest associations were observed for lung cancer, where minimally adjusted models showed a more than eightfold hazard ratio for top vs. bottom quartiles of GDF-15 (HR_Q4-Q1_ = 8.14 [95% CI: 4.70, 14.09]), a more than fourfold hazard ratio for top vs bottom quartiles of CRP (HR_Q4-Q1_ = 4.15 [2.55, 6.75]), and a more than threefold hazard ratio for top vs bottom quartiles of HbA1c (HR_Q4-Q1_ = 3.28 [2.13, 5.07]). Each of these associations with lung cancer risk were strongly attenuated, however, when models were additionally adjusted for smoking status and educational level (in addition to alcohol consumption for GDF-15) and further covariates in model 1. For NT-proBNP, HR associations (for breast, prostate, and colorectal cancer) were of moderate magnitude, with highest hazard ratio of 2.2 for top vs. bottom quartiles (colorectal cancer), and for this biomarker there was no change in HR estimates after additional covariate adjustments. Interestingly, levels of GDF-15 showed a significant inverse association with risk of prostate cancer. Cystatin-C levels showed no association with risks of prostate, lung or colorectal cancer risk, but showed a borderline significant inverse association for breast cancer in the multivariable-adjusted model.

Analyses among never, former and current-smokers showed statistically significant heterogeneity in biomarker risk associations only for HbA1C with risks of breast (p-heterogeneity = 0.01) and colorectal (p-heterogeneity = 0.02) cancer; where significant and moderately strong associations were observed only in never smokers (Supplemental Table S1). On the other hand, for lung cancer, marker associations were only statistically significant in current smokers; however, heterogeneity tests were non-significant, suggesting that absence of significance in never and former smokers might be linked to the limited number of lung cancer cases in these subgroups. Furthermore, although heterogeneity tests were not significant, the direct associations of NT-proBNP with colorectal and breast cancer risk were of a stronger magnitude or statistically significant exclusively among never smokers, respectively (Supplemental Table S1).

For breast cancer, further exploratory analyses by ER, PR and HER receptor status and by age at tumor diagnosis showed significant associations of HbA1c especially with risk of Her2 + cancers (p-heterogeneity = 0.04), and of tumors diagnosed after age 55, although no significant heterogeneity was observed (Supplemental Table S2). No statistically significant heterogeneity was observed for the associations of NT-proBNP with breast and prostate cancer risk, according to tumor subtypes, grade and age at diagnosis (Supplemental Table S2). The inverse association between GDF-15 and prostate cancer risk was more pronounced for high-grade disease, with a borderline significant heterogeneity (p-heterogeneity = 0.05).

Findings were unchanged when cases diagnosed within the first 2 years of follow-up were excluded. Only the association of cystatin-C with risk of stroke became non-significant (Supplemental Table S3).

When the five markers were examined simultaneously, in a mutually adjusted manner, MI risk was associated with levels of NT-proBNP, HbA1C, CRP and cystatin-C. As for stroke, associated biomarkers were GDF-15, NT-proBNP and CRP. By contrast, the only biomarkers associated with breast cancer risk were NT-proBNP and HbA1C, whereas only GDF-15 (negative) and NT-proBNP (positive) were associated with prostate cancer, only GDF-15 and HbA1C with lung cancer risk, and only NT-proBNP with colorectal cancer risk. Models combining these biomarkers, weighted by their effect sizes in the mutually adjusted models, and adjusted for BMI, lifetime alcohol consumption, smoking, physical activity level, educational level, baseline self-reported diabetes, and baseline self-reported hypertension, showed moderately strong associations with cancer and CVD risk, ranging from HR_Q4-Q1_ = 1.78 [1.36, 2.34] for breast cancer (combining NT-proBNP and HbA1C), to HR_Q4-Q1_ = 2.87 [2.15, 3.83] for MI (combining NT-proBNP, HbA1C, CRP and cystatin-C) (Table 4).

**Table 4:** Associations between mutually adjusted models, multi-marker combination and cancer and CVD risk, EPIC-Heidelberg (n = 7767)

	GDF-15		NT-proBNP		HbA1C		CRP		Cystatin-C	
	HR (95%CI)	p-value	HR (95%CI)	p-value	HR (95%CI)	p-value	HR (95%CI)	p-value	HR (95%CI)	p-value
Mutually adjusted models										
Breast cancer	1.00 (0.88;1.15)	0.94	1.18 (1.10;1.26)	< .0001	1.89 (1.15;3.13)	0.01	1.01 (0.96;1.07)	0.63	0.88 (0.76;1.01)	0.07
Prostate cancer	0.72 (0.57;0.90)	0.004	1.19 (1.11;1.27)	< .0001	1.48 (0.98;2.22)	0.06	1.06 (1.00;1.13)	0.06	1.03 (0.90;1.19)	0.65
Lung cancer	1.49 (1.24;1.80)	< .0001	1.05 (0.95;1.15)	0.37	2.23 (1.12;4.44)	0.02	1.08 (0.98;1.19)	0.11	0.99 (0.82;1.20)	0.92
Colorectal cancer	0.85 (0.67;1.09)	0.21	1.20 (1.10;1.31)	< .0001	1.28 (0.74;2.21)	0.38	1.05 (0.96;1.14)	0.28	1.00 (0.84;1.19)	0.99
MI	1.11 (0.95;1.30)	0.18	1.15 (1.08;1.23)	< .0001	1.89 (1.26;2.81)	0.002	1.14 (1.08;1.21)	< .0001	1.14 (1.01;1.28)	0.03
Stroke	1.32 (1.15;1.51)	0.0001	1.13 (1.06;1.21)	0.0001	1.19 (0.80;1.78)	0.3903	1.10 (1.04;1.16)	0.0005	1.08 (0.96;1.21)	0.21

	Q1	Q2	Q3	Q4	p-trend
	HR (95%CI)	HR (95%CI)	HR (95%CI)	HR (95%CI)	
Multi-marker combination					
Breast cancer	Ref	1.59 (1.23;2.07)	1.61 (1.23;2.10)	1.78 (1.36;2.34)	< .0001
Prostate cancer	Ref	1.48 (1.11;1.97)	1.61 (1.22;2.13)	1.83 (1.39;2.42)	< .0001
Lung cancer	Ref	1.05 (0.55;1.99)	1.80 (0.99;3.28)	2.44 (1.33;4.48)	0.002
Colorectal cancer	Ref	1.76 (1.17;2.65)	1.52 (1.00;2.32)	2.22 (1.50;3.28)	< .0001
MI	Ref	1.74 (1.30;2.32)	2.10 (1.57;2.82)	2.87 (2.15;3.83)	< .0001
Stroke	Ref	1.32 (1.00;1.74)	1.79 (1.37;2.35)	2.44 (1.86;3.20)	< .0001

HR: Hazard Ratio, CI: Confidence interval, MI: Myocardial infarction

Models were cause-specific, stratified for age (5-y category), adjusted for age (as timescale), sex (except for breast and prostate cancer), BMI, lifetime alcohol consumption, smoking status (never, long time quitters, short time quitters, current light, and current heavy smokers), physical activity level, educational level, baseline self-reported diabetes (for GDF-15 and HbA1C), baseline self-reported hypertension (for myocardial infarction and stroke), and mutually adjusted for the 4 other biomarkers (except for the models with the multi-marker scores)

Continuous HR for one unit increment in log-2 based biomarker = change in hazard associated with a doubling of biomarker concentration

Quartiles of the combination index constructed by the sum of each significant biomarker in the mutually adjusted model, weighted by its corresponding beta-coefficient (w); as follows: Breast cancer: NT-proBNP (w = 0.16403), HbA1C (w = 0.6387); Prostate cancer: GDF-15 (w = -0.33278), NT-proBNP (w = 0.17026); Lung cancer: GDF-15 (w = 0.39846), HbA1C (w = 0.80369); Colorectal cancer: NT-proBNP (w = 0.1834); MI: NT-proBNP (w = 0.14331), HbA1C (w = 0.63457), CRP (w = 0.1356), Cystatin-C (w = 0.12972); Stroke: GDF-15 (w = 0.27614), NT-proBNP (w = 0.12693), CRP (w = 0.09422). HRs were corrected to match case-cohort design using inverse subcohort sampling probability weighting (ISSP)

## Discussion

In this prospective analysis, we found risk associations for both CVD (MI, stroke) and cancer with five ageing-related biomarkers that were pre-selected on the basis of their measurement reliability, relevance to biological ageing, and ability to predict all-cause mortality. All five markers showed associations with long-term risk of CVD (NT-proBNP, CRP, HbA1C and Cystatin-C for MI; GDF-15, NT-proBNP and CRP for stroke). Regarding cancer, increased risks were observed especially among individuals who had higher blood concentrations of NT-proBNP (breast, prostate, colorectum), HbA1C (lung, colorectum, breast), and CRP (lung, colorectum), whereas higher GDF-15 levels were associated with an increased risk of lung cancer and a reduced risk of prostate cancer.

NT-proBNP was associated with breast, prostate, colorectal cancer and CVD risks. HbA1C was associated with breast, lung and colorectal (among never smokers) cancer and MI risks. CRP was associated with lung cancer and CVD risks. Cystatin-C was inversely associated with breast cancer risk and directly associated with CVD risk. For each of the chronic disease outcomes, combining the markers showed HRs between 1.78 (breast cancer) and 2.87 (MI) comparing highest to lowest quartiles, after adjustment for smoking, BMI, alcohol consumption, physical activity, and self-reported diabetes or hypertension.

Our findings for MI and stroke are consistent with those from previous studies and published meta-analyses [24–28], with very similar effect sizes for CRP [27], NT-proBNP [25], and GDF-15 [24]. A prospective study by Ho et al*.* used targeted proteomics to identify strong predictors of CVD and mortality, covering 85 pre-selected protein markers. In mutually adjusted analyses, GDF-15 was the only biomarker associated with CVD risk, whereas twelve further ones were associated with all-cause mortality, among which GDF-15, NT-proBNP, and Cystatin-C, and six (among which NT-proBNP and GDF-15) were associated specifically with CVD death [22]. Another prospective study by Daniels et al*.* [23] also found that GDF-15 and NT-proBNP, but not CRP, were independent predictors of overall and cardiovascular mortality, GDF-15 being the strongest for overall mortality and NT-proBNP for cardiovascular mortality. Participants in the highest quartiles of both biomarkers had a significantly higher risk of overall mortality (HR = 2.6 [2.0, 3.5]) compared to those in the lowest quartiles of both biomarkers. Our findings that all our five candidates are independent predictors for CVD risk (NT-proBNP, HbA1C, CRP and Cystatin-C for MI, and GDF-15, NT-proBNP and CRP for stroke) are consistent with these previous studies, and we also found a similarly strong association of marker combinations with risks of both MI (HR_Q4-Q1_ = 2.87, after adjustment for other established CVD risk factors) and stroke (HR_Q4-Q1_ = 2.44).

Regarding cancer, our findings for CRP and HbA1C are also quite in line with those from previous prospective studies. For CRP, previous prospective studies have shown increased risks of colorectal cancer [32], and of lung cancer among ever smokers [29, 30, 50], in association with higher blood levels. Likewise, previous studies have also found an increased risks of colorectal cancer [34, 35], and again of lung cancer among smokers [34, 35], for individuals who had elevated HbA1C, even within the normo-glycemic range. For breast cancer, by contrast, findings from previous studies have been inconsistent [34, 35], possibly because of heterogeneities in risk factor associations with breast cancer developing before or after menopause, or depending tumor sub-types.

Novel observations from our study are the increased risk of lung cancer, and the reduced risk of prostate cancer, among individuals who had higher blood levels of GDF-15. The association with lung cancer in part appeared confounded by smoking, but persisted after smoking was adjusted for (HR = 2.73 after adjustment for smoking and other confounders vs HR = 8.14 in models adjusted only for age and sex). Other studies have found higher levels of circulating GDF-15 in patients with pulmonary fibrosis [51] – a condition associated with high lung cancer risk [52]. For prostate cancer, the inverse association of risk with GDF-15 appeared more pronounced for high-grade than low-grade disease. These findings are consistent with those from a cross-sectional study [53] showing that prostate cancer cases had lower GDF-15 levels than men with benign prostatic hyperplasia. The latter study, however, showed that patients with higher Gleason scores had higher GDF-15 levels, unlike our findings showing a stronger inverse association for high-grade prostate cancer. Our findings suggested no association between circulating GDF-15 with breast or colorectal cancer; however, an independent prospective study showed a positive association between GDF-15 levels and colorectal cancer risk [40]. It is worth mentioning that two small prospective studies, one among diabetics and one among elderly individuals, found positive associations between higher levels of GDF-15 and overall cancer risk [37, 39]; these studies, however, were too small to examine relative risks for individual cancer types.

For NT-proBNP, our study showed for the first time in the general population moderately strong associations between higher blood levels and increased risks of breast, prostate and colorectal cancer. These findings are consistent with those from a small prospective study (n = 699 and 24 cancer cases) in coronary disease patients, linking NT-proBNP with overall cancer risk [42], and support findings from a retrospective study showing that BNP levels are elevated in cancer patients [54]. We did not observe an association with lung cancer, in contrary to findings from one cross-sectional study suggesting that lung cancer patients were more likely to have elevated NT-proBNP levels [55].

For cystatin-C our data suggest a possible inverse association with breast cancer risk, although this association was statistically significant only in multi-variate adjusted risk models or when cases diagnosed within the first 2 years were excluded. However, our data showed no evidence for any further associations of cystatin-C with risk of cancers of the colorectum, lung or prostate. We are not aware of other studies, so far, that prospectively examined the association of cystatin-C in initially cancer free subjects with later cancer risk.

To our knowledge, this study is the first to assess how combinations of these five biomarkers associate with cancer incidence. Daniels et al*.* found that in a mutually adjusted model, GDF-15 was the only predictor of cancer-specific mortality [23]. Our results suggested that NT-proBNP and HbA1C combined together, show a strong joint association with breast cancer incidence, while combining GDF-15 and HbA1C is strongly associated with lung cancer incidence. On the other hand, having simultaneously high levels of NT-proBNP and low levels of GDF-15 is associated with a 83% increase in prostate cancer risk.

Recent studies showed that biological ageing relies on several pillars, that might surprisingly interconnect [3, 15, 56], among which metabolism, inflammation, and adaptation to stress. Previous research suggests that each of our selected biomarkers might reflect one or several pathophysiological pathways underlying biological ageing and age-related diseases: GDF-15 has been described as a strong biomarker for biological ageing [57]. Mitochondrial dysfunction (strongly linked to ageing) in animal models increased GDF-15 levels; this trend was also observed in humans with mitochondrial disease, possibly through impaired calcium homeostasis and excessive oxidative stress and in older than in younger persons, potentially as a response to impaired calcium homeostasis and excessive oxidative stress [58, 59]. NT-proBNP, released from cardiomyocytes undergoing wall stress or ischemia and, well-known for indicating cardiovascular health, was suggested to be a strong indicator of biological ageing [15, 60] and might be stimulated by several pro-inflammatory cytokines, including tumor necrosis factor-α and some interleukins [42]. HbA1C is, in addition to its role in diabetes diagnosis, a marker of metabolically unhealthy biological ageing [61], and CRP reflects chronic inflammation linked to biological ageing [27]. Last, cystatin-C might, in addition to its role in kidney function, mediate an increase in other risk factors for ageing, such as anemia, insulin resistance and inflammation [62]. The five biomarkers might therefore reflect a systemic state of cellular or functional ageing. Indeed, moderate to strong correlations with chronological age have been described for these markers, especially GDF-15 (average age = 71 ± 11 years old) [23], NT-proBNP (average age = 68 ± 8 years old) [63] and cystatin-C (average age = 72 ± 4 years old) [62]. These correlations were weaker in our cohort (R^2^ ranged between 0.02 for CRP and 0.13 for GDF-15 and CRP); however, our participants were younger at the time of cohort enrollment and blood donation. Interestingly, participants above 50 years old in our cohort (55 for NT-proBNP) had clearly higher levels than younger participants (Table 2); which possibly suggests that the analyzed biomarkers might begin to show a stronger discriminatory ability of unhealthy ageing above the age of 50. While some combinations of these biomarkers have been shown to be strongly associated with mortality and cardiovascular health, our study was the first to show that the biomarkers might have a predictive capacity for cancer incidence. In regard to the complex process of transformation to malignancy, recent studies hypothesize that, independently of chronological age, some ageing-associated changes in the cellular microenvironment (such as increased inflammation or decreased immunity) might be required for carcinogenesis [64]. It is also unclear whether the biomarkers used in the present study are themselves biological mediators of effect, or whether they merely reflect the effects of other unmeasured biological factors or functional states; which might explain the moderate correlations with age. Indeed, Mendelian Randomization studies have shown that some of our biomarkers (GDF-15, cystatin-C) were not causally associated with cancer and CVD risks, respectively [38, 41, 65].

Biological ageing is surely influenced by genetic factors, however it can still be delayed or targeted by environmental influences [64], including lifestyle changes [7, 8] or pharmacological interventions [9]. Correlations between our selected biomarkers and lifestyle factors were relatively moderate: GDF-15 showed the strongest correlation with smoking (Spearman partial coefficient with number of pack-years and duration of smoking = 0.26 and 0.25, respectively, p < 0.001) and CRP was the only candidate with a moderate correlation with adiposity (Spearman partial coefficient with BMI = 0.37, p < 0.001). Therefore, even though our findings support the usefulness of combining these biomarkers to identify participants at higher NCD risk, our data provide no evidence on how healthier lifestyles might help improve the levels of these biomarkers, and thus, whether these biomarkers would help evaluate the efficacy of lifestyle prevention strategies. Further studies in other populations including younger and older adults are needed to investigate the associations between these biomarkers and lifestyle factors.

### Strengths and limitations

This study had several strengths. It was the first study to assess the associations between combinations of biomarkers linked to biological ageing (GDF-15, NT-proBNP, HbA1C, CRP, cystatin-C) and cancer risk. The present analyses were well-powered and comprehensively adjusted for potential confounders. Our study included healthy participants from the general population, free of major cardiovascular conditions or cancer at baseline. This has allowed detecting possible associations, before diagnosis was made or symptoms appear. We had data about histological grading for prostate cancer and breast tumor subtypes, as well as a relatively long follow-up duration (median 10y): even though this enabled us to investigate long term associations, a dilution effect could not be entirely ruled out. Nevertheless, this could have led to a non-differential measurement error (identically in cases and non-cases), most probably leading to an underestimation of the observed associations. Moreover, the risk of residual confounding, linked either to unmeasured factors (e.g., family history of diseases, blood pressure, some environmental exposures) or to imperfections in data collection (for smoking for instance) cannot be totally excluded despite adjusting for a 5-category smoking variable. However, the majority of associations with cancer risk were also found in never smokers. Moreover, even if these markers have shown strong associations with chronological age and mortality risk in previous studies, they cannot be interpreted as signatures of biological ageing. In addition, the multi-marker combination we developed was not cross-validated; however, it was not intended for further replication, or clinical practice, but it aimed to examine whether these markers, reflecting different dimensions of ageing hallmarks (inflammation, mitochondrial dysfunction, metabolic and functional ageing), jointly show stronger associations with cancer and CVD risk, in our study population, than considering each of them individually. Furthermore, even though these five markers were related to ageing, any direct inference of the observed associations to a specific single dimension of biological ageing is not straightforward, and should be interpreted with caution, as these markers are not specific to ageing, and could reflect other causal pathways (including for instance cellular stress, cardiovascular health, glucose intolerance, inflammation, or renal dysfunction) and as biological ageing covers a wide array of mechanisms and pathways. Last, the number of cases might have been limited to detect associations in stratified and tumor subtypes/grades models, leading to a weaker statistical power in some of these analyses.

## Conclusions

This study allowed us to have an umbrella view of the associations between five biomarkers linked to biological ageing (GDF-15, NT-proBNP, HbA1C, CRP, cystatin-C) and cancer and CVD risks. While all of them were independently associated with CVD risk, we observed for the first time that combining NT-proBNP and HbA1C associates strongly with breast cancer risk, GDF-15 and NT-proBNP with prostate cancer risk, and GDF-15 and HbA1C with lung cancer risk. If confirmed in other studies and settings, these findings open up the possibility of using combinations of biological ageing markers to predict cancer, CVD risk and mortality, which might enable risk stratification to identify people at high risk of NCDs.

## Supplementary Information

Below is the link to the electronic supplementary material.

Supplementary file1 (DOCX 692 kb)

## Data Availability

EPIC–Heidelberg was launched in the 1990s. Unlike in new studies run today, public access to data from the EPIC population was not part of the study protocol at that time. Thus, the data protection statement and informed consent of the EPIC participants do not cover the provision of data in public repositories. Nevertheless, we are open to providing our dataset upon request for (a) statistical validation by reviewers and (b) pooling projects under clearly defined and secure conditions and based on valid data transfer agreements.

## Declarations

### Disclaimer

None of the funders had any role in the study design; in collection, analysis, and interpretation of data; in the writing of the report; and in the decision to submit the manuscript for publication.
