# Supplementary material for: Ageing-related markers and risks of cancer and cardiovascular disease: a prospective study in the EPIC-Heidelberg cohort
Source: Eur J Epidemiol. 2021 Dec 22;37(1):49–65. doi: 10.1007/s10654-021-00828-3 (PMC8791871; doi:10.1007/s10654-021-00828-3)
Supplement: Supplementary file 1 — Supplementary file1 (DOCX 692 kb) [file 10654_2021_828_MOESM1_ESM.docx]

**Biomarkers of biological ageing and risks of cancer and cardiovascular disease in the EPIC-Heidelberg cohort**

Bernard Srour*, Rudolf Kaaks, Theron Johnson, Lucas Cory Hynes, Tilman Kühn, Verena Katzke**

[*b.srour@dkfz-heidelberg.de](mailto:*b.srour@dkfz-heidelberg.de)

[**v.katzke@dkfz-heidelberg.de](mailto:**v.katzke@dkfz-heidelberg.de)

*SUPPLEMENTAL MATERIAL*


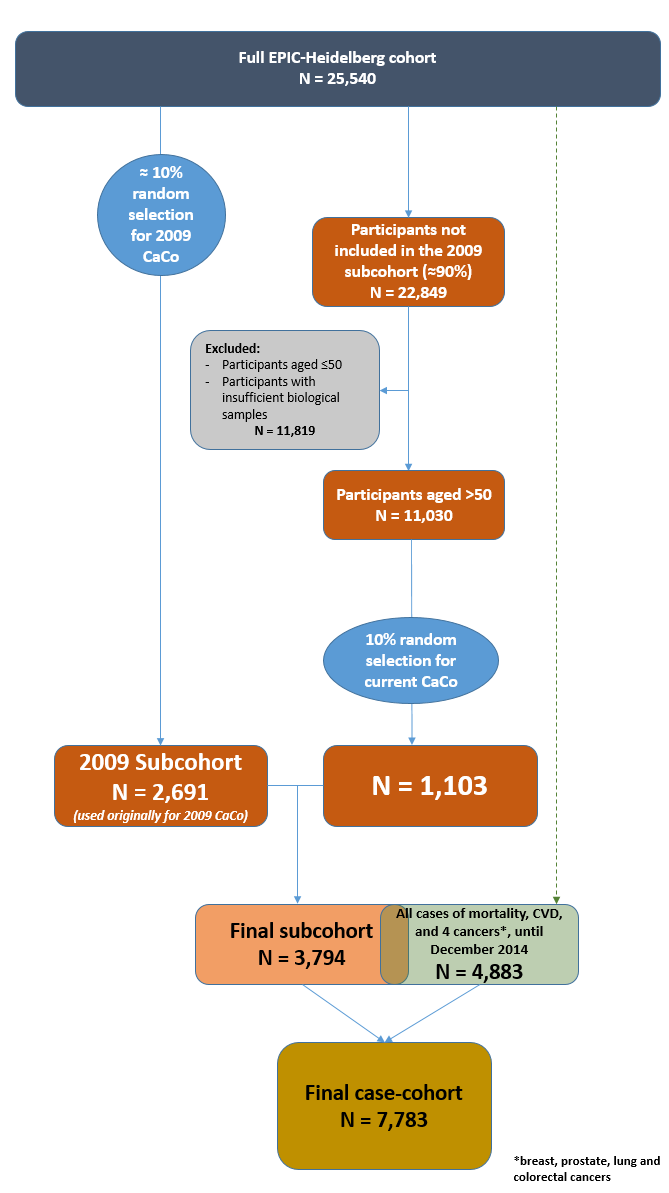


**Figure S1 - Flowchart for sample selection, EPIC-Heidelberg case-cohort, 1994-2014**


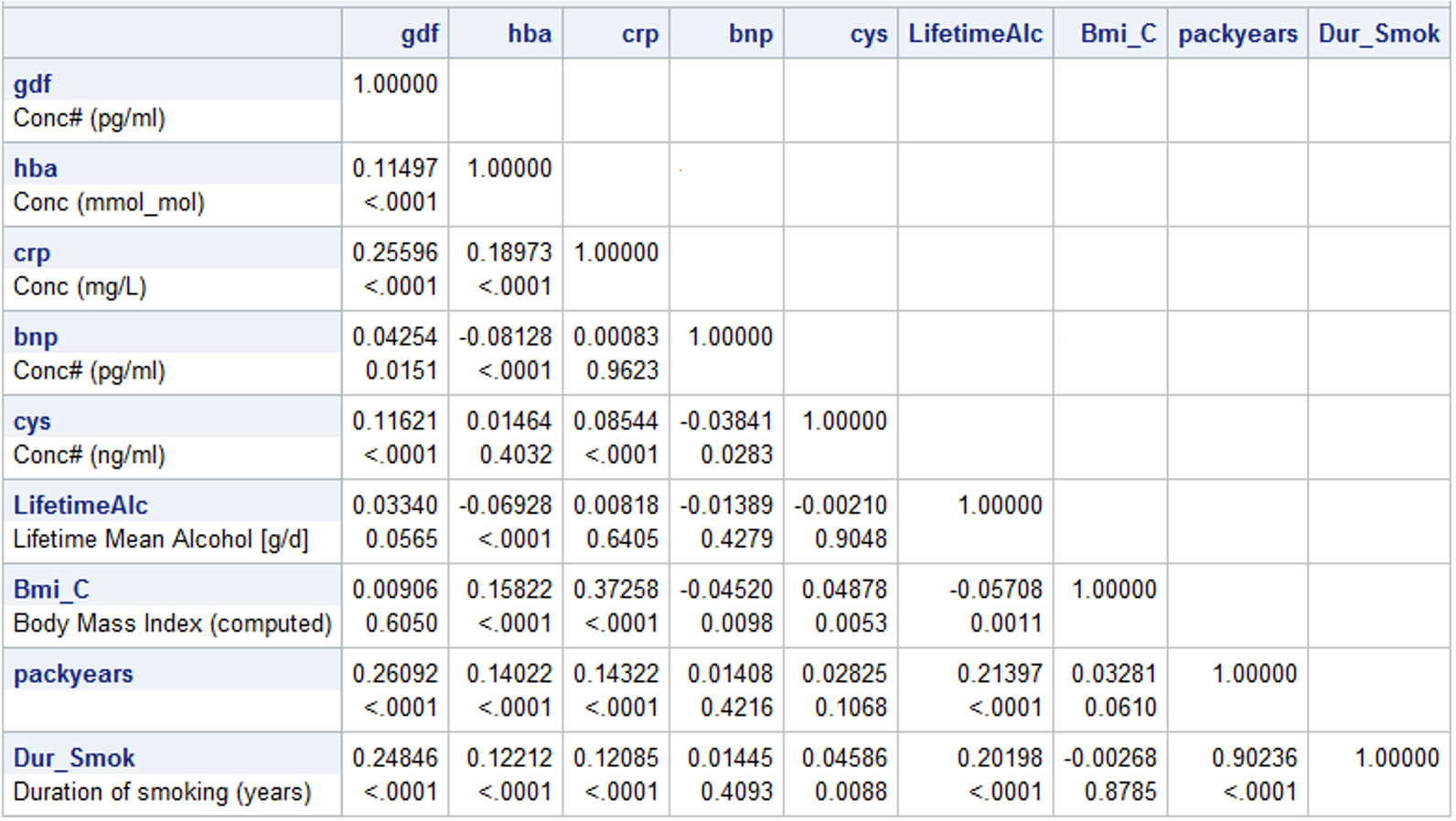


**Figure S2 – Spearman Partial Correlation Coefficients after adjustment for age and sex in the subcohort, EPIC-Heidelberg, n=3,792.**

**Table S1 - Hazard ratios and 95%CI for associations between GDF-15, NT-proBNP, HBA1C, CRP and Cystatin-C with risks of cancer and cardiovascular diseases, separately in never and ever (former and current) smokers, EPIC-Heidelberg case-cohort, n = 7,767**

|  | **Q1** | **Q2** | **Q3** | **Q4** | **P-trend** | **Continuous** | **P-value** |
| --- | --- | --- | --- | --- | --- | --- | --- |
|  |  |  |  |  |  |  |  |
| **GDF-15** | | | | | | | |
| **Breast cancer** |  |  |  |  |  |  |  |
| n cases never smokers | 73 | 90 | 78 | 79 |  |  |  |
| Model 2 | Ref | 1.22 (0.81,1.82) | 1.10 (0.72,1.68) | 1.17 (0.75,1.81) | 0.67 | 1.03 (0.83,1.28) | 0.77 |
| n cases former smokers | 42 | 45 | 56 | 45 |  |  |  |
| Model 2 | Ref | 1.00 (0.61,1.63) | 1.32 (0.80,2.17) | 1.18 (0.69,2.00) | 0.49 | 1.00 (0.80,1.25) | 0.99 |
| n cases current smokers | 31 | 34 | 21 | 29 |  |  |  |
| Model 2 | Ref | 1.22 (0.67,2.22) | 0.89 (0.46,1.71) | 1.50 (0.75,2.99) | 0.32 | 1.09 (0.78,1.53) | 0.62 |
| **Prostate cancer** |  |  |  |  |  |  |  |
| n cases never smokers | 48 | 44 | 79 | 55 |  |  |  |
| Model 2 | Ref | 0.66 (0.40,1.07) | 0.95 (0.60,1.51) | 0.55 (0.34,0.92) | 0.04 | 0.64 (0.45,0.91) | 0.01 |
| n cases former smokers | 56 | 67 | 69 | 70 |  |  |  |
| Model 2 | Ref | 0.98 (0.64,1.49) | 0.83 (0.54,1.26) | 0.80 (0.51,1.26) | 0.29 | 0.87 (0.60,1.26) | 0.45 |
| n cases current smokers | 21 | 20 | 24 | 24 |  |  |  |
| Model 2 | Ref | 0.94 (0.47,1.88) | 0.95 (0.45,2.01) | 1.22 (0.60,2.49) | 0.53 | 1.06 (0.69,1.62) | 0.80 |
| **Lung cancer** |  |  |  |  |  |  |  |
| n cases never smokers | 1 | 4 | 4 | 7 |  |  |  |
| Model 2 | Ref | 3.14 (0.49,19.91) | 2.69 (0.43,16.81) | 4.81 (0.76,30.27) | 0.18 | 1.66 (0.84,3.27) | 0.15 |
| n cases former smokers | 10 | 12 | 14 | 19 |  |  |  |
| Model 2 | Ref | 1.00 (0.41,2.42) | 0.89 (0.37,2.15) | 0.95 (0.38,2.34) | 0.95 | 1.06 (0.66,1.69) | 0.82 |
| n cases current smokers | 13 | 23 | 43 | 52 |  |  |  |
| Model 2 | Ref | 1.71 (0.80,3.65) | 3.18 (1.53,6.59) | 4.40 (2.15,9.00) | <.001 | 1.95 (1.46,2.60) | <.001 |
| **Colorectal cancer** |  |  |  |  |  |  |  |
| n cases never smokers | 16 | 21 | 27 | 24 |  |  |  |
| Model 2 | Ref | 0.98 (0.48,1.98) | 1.16 (0.55,2.46) | 0.82 (0.37,1.83) | 0.55 | 0.87 (0.57,1.33) | 0.53 |
| n cases former smokers | 21 | 35 | 35 | 27 |  |  |  |
| Model 2 | Ref | 1.42 (0.80,2.52) | 1.16 (0.64,2.09) | 0.81 (0.41,1.59) | 0.21 | 0.81 (0.53,1.24) | 0.33 |
| n cases current smokers | 11 | 17 | 16 | 18 |  |  |  |
| Model 2 | Ref | 1.42 (0.62,3.26) | 1.08 (0.46,2.55) | 1.29 (0.54,3.07) | 0.60 | 1.03 (0.64,1.65) | 0.90 |
| **Mycordial Infarction** |  |  |  |  |  |  |  |
| n cases never smokers | 30 | 44 | 71 | 62 |  |  |  |
| Model 2 | Ref | 1.07 (0.65,1.78) | 1.47 (0.90,2.39) | 0.96 (0.57,1.60) | 0.52 | 1.00 (0.74,1.35) | 0.99 |
| n cases former smokers | 40 | 50 | 68 | 93 |  |  |  |
| Model 2 | Ref | 1.20 (0.74,1.92) | 1.47 (0.92,2.35) | 2.03 (1.25,3.27) | <.001 | 1.43 (1.09,1.88) | 0.01 |
| n cases current smokers | 48 | 67 | 59 | 76 |  |  |  |
| Model 2 | Ref | 1.35 (0.85,2.13) | 1.19 (0.73,1.95) | 1.87 (1.18,2.98) | 0.02 | 1.51 (1.16,1.96) | <.001 |
| **Stroke** |  |  |  |  |  |  |  |
| n cases never smokers | 29 | 53 | 66 | 101 |  |  |  |
| Model 2 | Ref | 1.41 (0.86,2.31) | 1.56 (0.95,2.57) | 1.98 (1.21,3.24) | 0.01 | 1.50 (1.20,1.87) | <.001 |
| n cases former smokers | 41 | 47 | 77 | 108 |  |  |  |
| Model 2 | Ref | 1.05 (0.66,1.68) | 1.45 (0.93,2.26) | 1.89 (1.19,3.01) | <.001 | 1.45 (1.14,1.84) | <.001 |
| n cases current smokers | 39 | 47 | 55 | 61 |  |  |  |
| Model 2 | Ref | 1.09 (0.67,1.79) | 1.26 (0.76,2.08) | 1.48 (0.87,2.50) | 0.14 | 1.45 (1.08,1.96) | 0.01 |
| **NT-proBNP** | | | | | | | |
| **Breast cancer** |  |  |  |  |  |  |  |
| n cases never smokers | 51 | 77 | 83 | 99 |  |  |  |
| Model 2 | Ref | 1.62 (1.08,2.44) | 1.89 (1.26,2.84) | 2.19 (1.47,3.26) | <.001 | 1.29 (1.17,1.43) | <.001 |
| n cases former smokers | 31 | 54 | 39 | 56 |  |  |  |
| Model 2 | Ref | 1.91 (1.10,3.31) | 1.35 (0.75,2.43) | 1.88 (1.09,3.23) | 0.10 | 1.12 (0.99,1.27) | 0.07 |
| n cases current smokers | 22 | 29 | 37 | 24 |  |  |  |
| Model 2 | Ref | 1.23 (0.63,2.37) | 1.65 (0.84,3.23) | 0.99 (0.49,2.03) | 0.91 | 1.08 (0.91,1.28) | 0.38 |
| **Prostate cancer** |  |  |  |  |  |  |  |
| n cases never smokers | 38 | 55 | 56 | 66 |  |  |  |
| Model 2 | Ref | 1.65 (0.99,2.75) | 1.56 (0.94,2.60) | 1.65 (1.01,2.69) | 0.17 | 1.12 (1.00,1.24) | 0.05 |
| n cases former smokers | 32 | 52 | 81 | 84 |  |  |  |
| Model 2 | Ref | 1.54 (0.95,2.52) | 2.63 (1.64,4.20) | 2.38 (1.50,3.78) | <.001 | 1.24 (1.12,1.36) | <.001 |
| n cases current smokers | 20 | 21 | 20 | 24 |  |  |  |
| Model 2 | Ref | 1.09 (0.55,2.14) | 1.24 (0.58,2.63) | 1.50 (0.71,3.14) | 0.28 | 1.13 (0.94,1.35) | 0.20 |
| **Lung cancer** |  |  |  |  |  |  |  |
| n cases never smokers | 4 | 2 | 8 | 2 |  |  |  |
| Model 2 | Ref | 0.63 (0.12,3.49) | 2.61 (0.83,8.19) | 0.56 (0.10,3.01) | 0.63 | 1.12 (0.85,1.48) | 0.43 |
| n cases former smokers | 7 | 15 | 11 | 19 |  |  |  |
| Model 2 | Ref | 2.29 (0.90,5.84) | 1.49 (0.57,3.92) | 2.01 (0.81,4.97) | 0.43 | 1.12 (0.95,1.32) | 0.19 |
| n cases current smokers | 30 | 32 | 34 | 27 |  |  |  |
| Model 2 | Ref | 1.06 (0.59,1.88) | 1.19 (0.66,2.15) | 0.90 (0.50,1.63) | 0.58 | 1.00 (0.87,1.15) | 0.96 |
| **Colorectal cancer** |  |  |  |  |  |  |  |
| n cases never smokers | 10 | 26 | 16 | 32 |  |  |  |
| Model 2 | Ref | 2.92 (1.34,6.33) | 1.80 (0.79,4.11) | 3.15 (1.48,6.70) | 0.01 | 1.21 (1.05,1.40) | 0.01 |
| n cases former smokers | 18 | 28 | 28 | 39 |  |  |  |
| Model 2 | Ref | 1.54 (0.80,2.95) | 1.65 (0.87,3.12) | 2.14 (1.17,3.93) | 0.02 | 1.19 (1.05,1.36) | 0.01 |
| n cases current smokers | 12 | 17 | 13 | 18 |  |  |  |
| Model 2 | Ref | 1.80 (0.79,4.12) | 1.34 (0.57,3.12) | 1.86 (0.84,4.14) | 0.18 | 1.15 (0.96,1.38) | 0.14 |
| **Mycordial Infarction** |  |  |  |  |  |  |  |
| n cases never smokers | 32 | 45 | 64 | 56 |  |  |  |
| Model 2 | Ref | 1.53 (0.92,2.52) | 2.25 (1.39,3.63) | 1.66 (1.02,2.72) | 0.10 | 1.16 (1.05,1.28) | 0.01 |
| n cases former smokers | 43 | 55 | 52 | 83 |  |  |  |
| Model 2 | Ref | 1.27 (0.80,2.00) | 1.18 (0.75,1.86) | 1.88 (1.23,2.87) | <.001 | 1.20 (1.08,1.34) | <.001 |
| n cases current smokers | 60 | 58 | 51 | 73 |  |  |  |
| Model 2 | Ref | 1.06 (0.67,1.68) | 0.95 (0.59,1.51) | 1.62 (1.03,2.53) | 0.02 | 1.12 (0.99,1.28) | 0.08 |
| **Stroke** |  |  |  |  |  |  |  |
| n cases never smokers | 54 | 47 | 55 | 82 |  |  |  |
| Model 2 | Ref | 0.94 (0.60,1.46) | 1.06 (0.68,1.63) | 1.34 (0.90,1.98) | 0.06 | 1.13 (1.01,1.26) | 0.03 |
| n cases former smokers | 53 | 55 | 67 | 85 |  |  |  |
| Model 2 | Ref | 1.04 (0.68,1.59) | 1.23 (0.82,1.84) | 1.41 (0.94,2.09) | 0.04 | 1.13 (1.02,1.25) | 0.02 |
| n cases current smokers | 36 | 45 | 53 | 62 |  |  |  |
| Model 2 | Ref | 1.33 (0.80,2.19) | 1.49 (0.91,2.45) | 2.06 (1.26,3.36) | <.001 | 1.22 (1.06,1.39) | <.001 |
| **HbA1C** | | | | | | | |
| **Breast cancer** |  |  |  |  |  |  |  |
| n cases never smokers | 83 | 99 | 61 | 100 |  |  |  |
| Model 2 | Ref | 1.17 (0.83,1.65) | 1.26 (0.84,1.88) | 2.01 (1.37,2.94) | <.001 | 3.94 (1.72,9.05) | <.001 |
| n cases former smokers | 46 | 61 | 59 | 35 |  |  |  |
| Model 2 | Ref | 1.13 (0.72,1.79) | 1.30 (0.80,2.12) | 0.97 (0.55,1.70) | 0.91 | 1.09 (0.48,2.46) | 0.85 |
| n cases current smokers | 35 | 49 | 29 | 18 |  |  |  |
| Model 2 | Ref | 1.70 (0.94,3.08) | 1.24 (0.66,2.33) | 0.92 (0.43,1.98) | 0.86 | 0.66 (0.21,2.14) | 0.49 |
| **Prostate cancer** |  |  |  |  |  |  |  |
| n cases never smokers | 56 | 61 | 57 | 53 |  |  |  |
| Model 2 | Ref | 0.98 (0.63,1.52) | 1.10 (0.70,1.71) | 1.29 (0.80,2.10) | 0.27 | 1.69 (0.87,3.27) | 0.12 |
| n cases former smokers | 64 | 66 | 71 | 65 |  |  |  |
| Model 2 | Ref | 0.88 (0.60,1.31) | 1.40 (0.95,2.07) | 1.10 (0.70,1.74) | 0.30 | 1.12 (0.60,2.08) | 0.72 |
| n cases current smokers | 29 | 15 | 25 | 23 |  |  |  |
| Model 2 | Ref | 0.61 (0.29,1.28) | 0.74 (0.39,1.41) | 0.81 (0.36,1.81) | 0.61 | 0.94 (0.24,3.69) | 0.92 |
| **Lung cancer** |  |  |  |  |  |  |  |
| n cases never smokers | 6 | 1 | 5 | 5 |  |  |  |
| Model 2 | Ref | 0.13 (0.02,1.06) | 0.81 (0.21,3.08) | 0.62 (0.16,2.46) | 0.76 | 0.64 (0.11,3.54) | 0.60 |
| n cases former smokers | 7 | 13 | 19 | 16 |  |  |  |
| Model 2 | Ref | 1.47 (0.56,3.89) | 2.55 (1.00,6.50) | 1.80 (0.62,5.25) | 0.19 | 2.16 (0.62,7.51) | 0.23 |
| n cases current smokers | 24 | 28 | 56 | 33 |  |  |  |
| Model 2 | Ref | 1.56 (0.83,2.90) | 2.13 (1.19,3.82) | 2.16 (1.08,4.31) | 0.02 | 3.21 (1.23,8.39) | 0.02 |
| **Colorectal cancer** |  |  |  |  |  |  |  |
| n cases never smokers | 19 | 16 | 23 | 36 |  |  |  |
| Model 2 | Ref | 0.73 (0.37,1.44) | 1.45 (0.78,2.70) | 2.01 (1.08,3.73) | 0.01 | 2.58 (1.05,6.31) | 0.04 |
| n cases former smokers | 24 | 38 | 33 | 27 |  |  |  |
| Model 2 | Ref | 1.36 (0.79,2.32) | 1.54 (0.88,2.70) | 0.95 (0.47,1.93) | 0.99 | 0.87 (0.34,2.20) | 0.77 |
| n cases current smokers | 24 | 6 | 20 | 13 |  |  |  |
| Model 2 | Ref | 0.28 (0.11,0.72) | 0.65 (0.31,1.37) | 0.39 (0.16,0.94) | 0.08 | 0.40 (0.10,1.65) | 0.21 |
| **Mycordial Infarction** |  |  |  |  |  |  |  |
| n cases never smokers | 41 | 54 | 41 | 81 |  |  |  |
| Model 2 | Ref | 1.16 (0.74,1.81) | 1.11 (0.69,1.78) | 1.54 (0.96,2.46) | 0.09 | 2.23 (1.05,4.74) | 0.04 |
| n cases former smokers | 52 | 63 | 63 | 79 |  |  |  |
| Model 2 | Ref | 0.97 (0.64,1.47) | 1.41 (0.92,2.16) | 1.44 (0.90,2.29) | 0.06 | 1.56 (0.79,3.07) | 0.20 |
| n cases current smokers | 54 | 46 | 80 | 74 |  |  |  |
| Model 2 | Ref | 1.04 (0.64,1.67) | 1.52 (0.98,2.35) | 1.79 (1.08,2.98) | 0.02 | 2.10 (1.03,4.25) | 0.04 |
| **Stroke** |  |  |  |  |  |  |  |
| n cases never smokers | 48 | 74 | 54 | 70 |  |  |  |
| Model 2 | Ref | 1.33 (0.89,1.99) | 1.40 (0.91,2.15) | 1.23 (0.77,1.96) | 0.42 | 1.04 (0.56,1.92) | 0.90 |
| n cases former smokers | 58 | 62 | 65 | 82 |  |  |  |
| Model 2 | Ref | 0.85 (0.57,1.27) | 1.15 (0.77,1.74) | 1.20 (0.76,1.88) | 0.25 | 1.71 (0.84,3.49) | 0.14 |
| n cases current smokers | 53 | 39 | 58 | 50 |  |  |  |
| Model 2 | Ref | 0.90 (0.56,1.44) | 1.03 (0.65,1.62) | 1.14 (0.66,1.97) | 0.57 | 1.28 (0.56,2.92) | 0.55 |
| **CRP** | | | | | | | |
| **Breast cancer** |  |  |  |  |  |  |  |
| n cases never smokers | 77 | 82 | 84 | 75 |  |  |  |
| Model 2 | Ref | 1.21 (0.83,1.76) | 1.26 (0.86,1.86) | 1.23 (0.81,1.87) | 0.59 | 1.03 (0.95,1.11) | 0.49 |
| n cases former smokers | 42 | 43 | 57 | 45 |  |  |  |
| Model 2 | Ref | 1.04 (0.62,1.75) | 1.46 (0.87,2.46) | 1.23 (0.72,2.10) | 0.60 | 1.04 (0.94,1.15) | 0.47 |
| n cases current smokers | 35 | 29 | 21 | 27 |  |  |  |
| Model 2 | Ref | 1.01 (0.54,1.89) | 0.70 (0.34,1.41) | 0.94 (0.47,1.88) | 0.93 | 0.99 (0.86,1.14) | 0.89 |
| **Prostate cancer** |  |  |  |  |  |  |  |
| n cases never smokers | 41 | 57 | 64 | 62 |  |  |  |
| Model 2 | Ref | 1.21 (0.74,1.98) | 1.28 (0.79,2.08) | 1.21 (0.72,2.04) | 0.69 | 1.04 (0.94,1.15) | 0.48 |
| n cases former smokers | 68 | 56 | 75 | 61 |  |  |  |
| Model 2 | Ref | 0.78 (0.52,1.19) | 0.98 (0.66,1.47) | 0.85 (0.55,1.32) | 0.72 | 1.02 (0.92,1.13) | 0.71 |
| n cases current smokers | 10 | 26 | 30 | 22 |  |  |  |
| Model 2 | Ref | 2.52 (1.07,5.93) | 2.97 (1.28,6.92) | 2.31 (0.93,5.73) | 0.52 | 1.18 (1.01,1.38) | 0.03 |
| **Lung cancer** |  |  |  |  |  |  |  |
| n cases never smokers | 2 | 3 | 8 | 3 |  |  |  |
| Model 2 | Ref | 0.91 (0.12,6.61) | 2.50 (0.49,12.69) | 0.91 (0.14,6.11) | 0.66 | 0.99 (0.76,1.29) | 0.94 |
| n cases former smokers | 9 | 4 | 16 | 24 |  |  |  |
| Model 2 | Ref | 0.39 (0.12,1.33) | 1.41 (0.55,3.64) | 2.02 (0.76,5.34) | 0.03 | 1.18 (0.96,1.45) | 0.11 |
| n cases current smokers | 21 | 25 | 46 | 36 |  |  |  |
| Model 2 | Ref | 1.07 (0.54,2.11) | 2.07 (1.11,3.84) | 1.55 (0.80,2.98) | 0.19 | 1.17 (1.03,1.32) | 0.01 |
| **Colorectal cancer** |  |  |  |  |  |  |  |
| n cases never smokers | 15 | 19 | 24 | 28 |  |  |  |
| Model 2 | Ref | 1.04 (0.51,2.11) | 1.17 (0.58,2.34) | 1.34 (0.64,2.83) | 0.32 | 1.14 (0.98,1.32) | 0.09 |
| n cases former smokers | 20 | 26 | 39 | 27 |  |  |  |
| Model 2 | Ref | 1.17 (0.62,2.22) | 1.56 (0.85,2.88) | 1.04 (0.52,2.07) | 0.57 | 1.01 (0.88,1.16) | 0.91 |
| n cases current smokers | 13 | 8 | 25 | 15 |  |  |  |
| Model 2 | Ref | 0.56 (0.21,1.47) | 1.73 (0.78,3.85) | 0.89 (0.37,2.16) | 0.85 | 1.06 (0.87,1.29) | 0.55 |
| **Mycordial Infarction** |  |  |  |  |  |  |  |
| n cases never smokers | 29 | 37 | 48 | 89 |  |  |  |
| Model 2 | Ref | 0.97 (0.56,1.67) | 1.12 (0.67,1.87) | 1.88 (1.12,3.16) | <.001 | 1.15 (1.05,1.27) | 0.01 |
| n cases former smokers | 40 | 48 | 78 | 82 |  |  |  |
| Model 2 | Ref | 1.11 (0.69,1.78) | 1.77 (1.13,2.77) | 1.85 (1.17,2.92) | 0.01 | 1.17 (1.06,1.28) | <.001 |
| n cases current smokers | 26 | 48 | 82 | 89 |  |  |  |
| Model 2 | Ref | 1.78 (1.03,3.07) | 2.55 (1.50,4.34) | 2.78 (1.62,4.79) | <.001 | 1.26 (1.13,1.40) | <.001 |
| **Stroke** |  |  |  |  |  |  |  |
| n cases never smokers | 42 | 51 | 70 | 81 |  |  |  |
| Model 2 | Ref | 0.98 (0.62,1.56) | 1.12 (0.72,1.74) | 1.24 (0.78,1.97) | 0.32 | 1.06 (0.97,1.16) | 0.21 |
| n cases former smokers | 42 | 44 | 79 | 102 |  |  |  |
| Model 2 | Ref | 1.00 (0.63,1.59) | 1.60 (1.04,2.46) | 2.06 (1.33,3.20) | <.001 | 1.21 (1.11,1.32) | <.001 |
| n cases current smokers | 28 | 44 | 63 | 66 |  |  |  |
| Model 2 | Ref | 1.60 (0.92,2.76) | 2.21 (1.30,3.75) | 2.21 (1.27,3.84) | 0.05 | 1.17 (1.05,1.30) | <.001 |
| **Cystatin-C** | | | | | | | |
| **Breast cancer** |  |  |  |  |  |  |  |
| n cases never smokers | 78 | 92 | 69 | 77 |  |  |  |
| Model 2 | Ref | 1.23 (0.84,1.78) | 0.88 (0.58,1.32) | 1.05 (0.69,1.60) | 0.83 | 0.96 (0.79,1.18) | 0.72 |
| n cases former smokers | 54 | 48 | 44 | 41 |  |  |  |
| Model 2 | Ref | 0.85 (0.51,1.42) | 0.79 (0.46,1.37) | 0.70 (0.38,1.26) | 0.24 | 0.80 (0.60,1.06) | 0.12 |
| n cases current smokers | 40 | 32 | 23 | 20 |  |  |  |
| Model 2 | Ref | 0.72 (0.40,1.31) | 0.48 (0.24,0.94) | 0.49 (0.22,1.10) | 0.08 | 0.69 (0.47,1.03) | 0.07 |
| **Prostate cancer** |  |  |  |  |  |  |  |
| n cases never smokers | 49 | 63 | 50 | 60 |  |  |  |
| Model 2 | Ref | 1.07 (0.68,1.71) | 0.71 (0.44,1.14) | 0.80 (0.50,1.26) | 0.21 | 0.89 (0.71,1.11) | 0.30 |
| n cases former smokers | 44 | 77 | 55 | 78 |  |  |  |
| Model 2 | Ref | 1.43 (0.93,2.20) | 0.98 (0.62,1.55) | 1.29 (0.84,1.99) | 0.63 | 1.10 (0.89,1.36) | 0.38 |
| n cases current smokers | 18 | 19 | 22 | 29 |  |  |  |
| Model 2 | Ref | 0.97 (0.47,2.02) | 0.91 (0.44,1.91) | 1.39 (0.68,2.83) | 0.29 | 1.21 (0.89,1.66) | 0.23 |
| **Lung cancer** |  |  |  |  |  |  |  |
| n cases never smokers | 0 | 6 | 5 | 4 |  |  |  |
| Model 2 | - | - | - | - | - | 1.10 (0.70,1.73) | 0.68 |
| n cases former smokers | 16 | 9 | 15 | 14 |  |  |  |
| Model 2 | Ref | 0.47 (0.18,1.26) | 0.67 (0.28,1.59) | 0.53 (0.22,1.29) | 0.32 | 0.76 (0.50,1.15) | 0.19 |
| n cases current smokers | 28 | 32 | 37 | 32 |  |  |  |
| Model 2 | Ref | 1.24 (0.68,2.25) | 1.25 (0.69,2.25) | 1.25 (0.67,2.32) | 0.56 | 1.22 (0.94,1.58) | 0.13 |
| **Colorectal cancer** |  |  |  |  |  |  |  |
| n cases never smokers | 19 | 19 | 20 | 28 |  |  |  |
| Model 2 | Ref | 0.85 (0.44,1.64) | 0.82 (0.41,1.62) | 1.08 (0.56,2.06) | 0.65 | 0.98 (0.72,1.35) | 0.91 |
| n cases former smokers | 28 | 28 | 32 | 29 |  |  |  |
| Model 2 | Ref | 0.91 (0.51,1.62) | 0.99 (0.56,1.72) | 0.84 (0.47,1.51) | 0.65 | 0.96 (0.71,1.31) | 0.81 |
| n cases current smokers | 6 | 19 | 21 | 15 |  |  |  |
| Model 2 | Ref | 2.56 (0.97,6.74) | 2.54 (1.00,6.49) | 1.65 (0.62,4.38) | 0.96 | 1.05 (0.80,1.38) | 0.73 |
| **Mycordial Infarction** |  |  |  |  |  |  |  |
| n cases never smokers | 37 | 49 | 55 | 64 |  |  |  |
| Model 2 | Ref | 1.12 (0.69,1.80) | 1.09 (0.67,1.78) | 1.10 (0.67,1.80) | 0.79 | 1.10 (0.88,1.37) | 0.40 |
| n cases former smokers | 46 | 67 | 58 | 73 |  |  |  |
| Model 2 | Ref | 1.52 (0.99,2.35) | 1.38 (0.88,2.16) | 1.58 (1.02,2.47) | 0.14 | 1.22 (0.98,1.50) | 0.07 |
| n cases current smokers | 63 | 57 | 53 | 73 |  |  |  |
| Model 2 | Ref | 0.92 (0.58,1.43) | 0.76 (0.48,1.20) | 1.11 (0.71,1.73) | 0.57 | 1.15 (0.94,1.42) | 0.17 |
| **Stroke** |  |  |  |  |  |  |  |
| n cases never smokers | 48 | 61 | 67 | 69 |  |  |  |
| Model 2 | Ref | 1.02 (0.67,1.54) | 1.01 (0.67,1.52) | 0.92 (0.60,1.41) | 0.61 | 1.08 (0.88,1.32) | 0.46 |
| n cases former smokers | 46 | 77 | 71 | 75 |  |  |  |
| Model 2 | Ref | 1.49 (0.98,2.26) | 1.32 (0.86,2.02) | 1.31 (0.85,2.02) | 0.65 | 1.10 (0.91,1.34) | 0.33 |
| n cases current smokers | 36 | 46 | 63 | 57 |  |  |  |
| Model 2 | Ref | 1.21 (0.73,2.00) | 1.59 (0.98,2.58) | 1.49 (0.90,2.48) | 0.13 | 1.24 (0.99,1.54) | 0.06 |

Quartiles are based on the sex-specific distribution of the biomarker in the subcohort.
P-interaction respectively for breast, prostate, lung, colorectal cancers, MI and stroke were:
GDF-15: 0.53, 0.26, 0.86, 0.97, 0.19, 0.64
NT-proBNP: 0.06, 0.38, 0.68, 0.76, 0.70, 0.86
HbA1C: **0.01**, 0.43, 0.61, **0.02,** 0.05, 0.57
CRP: 0.81, 0.77, 0.22, 0.32, 0.33, 0.06
Cystatin-C: 0.11, 0.18**,** 0.23, 0.88, 0.71, 0.87.
HR for quartiles of cystatin-C and lung cancer in never smokers could not be computed due to absence of cases in Q1.
Model 2 is a cause-specific Cox model stratified for age (as 5-y categories), adjusted for age (timescale), sex, BMI, lifetime alcohol consumption, smoking status in former and current (long time quitters/short time quitters or current light/current heavy smokers), physical activity level, educational level, baseline self-reported diabetes (for GDF-15 and HbA1C), and baseline self-reported hypertension (for myocardial infarction and stroke).
Continuous HR for one unit increment in log-2 based biomarker = change in hazard associated with a doubling of biomarker concentration
HRs were corrected to match case-cohort design using inverse subcohort sampling probability weighting (ISSP)

**Table S2 - Hazard ratios and 95%CI for associations between GDF-15, NT-proBNP, HBA1C, CRP and Cystatin-C with risks of breast and prostate cancer according to tumour subtypes and grade, EPIC-Heidelberg case-cohort, n = 7,767**

|  | **Q1** | **Q2** | **Q3** | **Q4** | **P-trend** | **Continuous** | **P** |
| --- | --- | --- | --- | --- | --- | --- | --- |
|  |  |  |  |  |  |  |  |
| **GDF-15** | | | | | | | |
| **Breast cancer ER-** |  |  |  |  |  |  |  |
| Number of cases | 25 | 40 | 22 | 26 |  |  |  |
| Model 2 | Ref | 1.74 (1.01,3.02) | 1.04 (0.55,1.96) | 1.30 (0.68,2.49) | 0.87 | 1.00 (0.77,1.30) | 0.98 |
| **Breast cancer Her2+** |  |  |  |  |  |  |  |
| Number of cases | 26 | 29 | 22 | 18 |  |  |  |
| Model 2 | Ref | 1.37 (0.76,2.45) | 1.31 (0.68,2.53) | 1.16 (0.56,2.40) | 0.82 | 1.04 (0.79,1.36) | 0.80 |
| **Luminal breast cancer** |  |  |  |  |  |  |  |
| Number of cases | 82 | 106 | 98 | 84 |  |  |  |
| Model 2 | Ref | 1.23 (0.88,1.70) | 1.18 (0.83,1.68) | 1.15 (0.79,1.66) | 0.68 | 1.02 (0.87,1.21) | 0.78 |
| **Triple negative BC** |  |  |  |  |  |  |  |
| Number of cases | 10 | 16 | 13 | 15 |  |  |  |
| Model 2 | Ref | 1.57 (0.67,3.65) | 1.30 (0.52,3.24) | 1.49 (0.59,3.76) | 0.58 | 1.01 (0.80,1.28) | 0.93 |
| **Breast cancer <55 yo** |  |  |  |  |  |  |  |
| Number of cases | 70 | 54 | 35 | 23 |  |  |  |
| Model 2 | Ref | 1.29 (0.85,1.94) | 1.46 (0.90,2.36) | 1.26 (0.73,2.19) | 0.29 | 1.11 (0.93,1.32) | 0.24 |
| **Breast cancer ≥55 yo** |  |  |  |  |  |  |  |
| Number of cases | 75 | 121 | 121 | 124 |  |  |  |
| Model 2 | Ref | 1.13 (0.81,1.58) | 1.02 (0.73,1.44) | 1.11 (0.78,1.58) | 0.74 | 0.95 (0.79,1.13) | 0.54 |
| **Low-grade Pca** |  |  |  |  |  |  |  |
| Number of cases | 84 | 93 | 111 | 93 |  |  |  |
| Model 2 | Ref | 0.89 (0.64,1.24) | 0.91 (0.65,1.27) | 0.80 (0.56,1.16) | 0.28 | 0.86 (0.67,1.10) | 0.24 |
| **High-grade Pca** |  |  |  |  |  |  |  |
| Number of cases | 46 | 42 | 45 | 29 |  |  |  |
| Model 2 | Ref | 0.70 (0.44,1.11) | 0.63 (0.40,1.01) | 0.43 (0.24,0.76) | 0.01 | 0.50 (0.33,0.75) | <.001 |
| **NT-proBNP** | | | | | | | |
| **Breast cancer ER-** |  |  |  |  |  |  |  |
| Number of cases | 20 | 30 | 26 | 34 |  |  |  |
| Model 2 | Ref | 1.46 (0.80,2.65) | 1.21 (0.65,2.24) | 1.67 (0.93,3.01) | 0.13 | 1.18 (1.01,1.36) | 0.03 |
| **Breast cancer Her2+** |  |  |  |  |  |  |  |
| Number of cases | 19 | 21 | 27 | 26 |  |  |  |
| Model 2 | Ref | 0.95 (0.49,1.84) | 1.21 (0.65,2.27) | 1.22 (0.65,2.28) | 0.40 | 1.12 (0.95,1.32) | 0.18 |
| **Luminal breast cancer** |  |  |  |  |  |  |  |
| Number of cases | 62 | 93 | 98 | 102 |  |  |  |
| Model 2 | Ref | 1.60 (1.12,2.30) | 1.75 (1.22,2.52) | 1.81 (1.27,2.59) | 0.005 | 1.18 (1.02,1.28) | <.001 |
| **Triple negative BC** |  |  |  |  |  |  |  |
| Number of cases | 7 | 18 | 10 | 16 |  |  |  |
| Model 2 | Ref | 2.65 (1.09,6.47) | 1.50 (0.56,4.03) | 2.52 (1.00,6.38) | 0.18 | 1.23 (0.99,1.53) | 0.06 |
| **Breast cancer <55 yo** |  |  |  |  |  |  |  |
| Number of cases | 29 | 61 | 48 | 44 |  |  |  |
| Model 2 | Ref | 1.13 (0.69,1.85) | 0.85 (0.51,1.43) | 1.01 (0.60,1.71) | 0.77 | 0.99 (0.86,1.14) | 0.87 |
| **Breast cancer ≥55 yo** |  |  |  |  |  |  |  |
| Number of cases | 74 | 100 | 111 | 135 |  |  |  |
| Model 2 | Ref | 1.77 (1.26,2.49) | 2.07 (1.47,2.91) | 2.25 (1.62,3.13) | <.001 | 1.26 (1.16,1.36) | <.001 |
| **Low-grade Pca** |  |  |  |  |  |  |  |
| Number of cases | 56 | 83 | 107 | 120 |  |  |  |
| Model 2 | Ref | 1.55 (1.08,2.25) | 2.05 (1.43,2.93) | 2.01 (1.42,2.85) | <.001 | 1.17 (1.09,1.25) | <.001 |
| **High-grade Pca** |  |  |  |  |  |  |  |
| Number of cases | 28 | 39 | 38 | 45 |  |  |  |
| Model 2 | Ref | 1.48 (0.88,2.47) | 1.45 (0.87,2.43) | 1.72 (1.04,2.87) | 0.08 | 1.17 (1.04,1.32) | 0.01 |
| **HbA1C** | | | | | | | |
| **Breast cancer ER-** |  |  |  |  |  |  |  |
| Number of cases | 29 | 26 | 35 | 27 |  |  |  |
| Model 2 | Ref | 1.41 (0.81,2.44) | 1.65 (0.98,2.80) | 1.53 (0.85,2.79) | 0.13 | 1.66 (0.61,4.57) | 0.32 |
| **Breast cancer Her2+** |  |  |  |  |  |  |  |
| Number of cases | 31 | 22 | 27 | 24 |  |  |  |
| Model 2 | Ref | 1.17 (0.65,2.09) | 1.23 (0.69,2.19) | 1.57 (0.85,2.89) | 0.17 | 3.26 (1.11,9.57) | 0.03 |
| **Luminal breast cancer** |  |  |  |  |  |  |  |
| Number of cases | 108 | 92 | 116 | 91 |  |  |  |
| Model 2 | Ref | 1.33 (0.98,1.81) | 1.35 (0.99,1.84) | 1.34 (0.95,1.89) | 0.11 | 1.79 (0.95,3.39) | 0.07 |
| **Triple negative BC** |  |  |  |  |  |  |  |
| Number of cases | 16 | 10 | 18 | 13 |  |  |  |
| Model 2 | Ref | 0.92 (0.41,2.04) | 1.32 (0.66,2.63) | 0.91 (0.38,2.17) | 0.96 | 0.56 (0.13,2.39) | 0.44 |
| **Breast cancer <55 yo** |  |  |  |  |  |  |  |
| Number of cases | 77 | 45 | 50 | 21 |  |  |  |
| Model 2 | Ref | 1.19 (0.78,1.81) | 1.55 (1.00,2.42) | 1.22 (0.67,2.22) | 0.17 | 1.70 (0.64,4.56) | 0.29 |
| **Breast cancer ≥55 yo** |  |  |  |  |  |  |  |
| Number of cases | 101 | 102 | 139 | 140 |  |  |  |
| Model 2 | Ref | 1.33 (0.97,1.81) | 1.27 (0.94,1.71) | 1.47 (1.07,2.01) | 0.04 | 1.83 (1.02,3.29) | 0.04 |
| **Low-grade Pca** |  |  |  |  |  |  |  |
| Number of cases | 114 | 72 | 102 | 97 |  |  |  |
| Model 2 | Ref | 1.11 (0.80,1.55) | 1.32 (0.97,1.78) | 1.23 (0.88,1.71) | 0.12 | 1.46 (0.93,2.29) | 0.10 |
| **High-grade Pca** |  |  |  |  |  |  |  |
| Number of cases | 51 | 29 | 35 | 48 |  |  |  |
| Model 2 | Ref | 1.00 (0.61,1.61) | 0.97 (0.61,1.54) | 1.42 (0.90,2.24) | 0.21 | 1.29 (0.62,2.70) | 0.50 |
| **CRP** | | | | | | | |
| **Breast cancer ER-** |  |  |  |  |  |  |  |
| Number of cases | 26 | 31 | 22 | 33 |  |  |  |
| Model 2 | Ref | 1.32 (0.76,2.29) | 0.98 (0.54,1.77) | 1.56 (0.87,2.81) | 0.18 | 1.05 (0.93,1.19) | 0.42 |
| **Breast cancer Her2+** |  |  |  |  |  |  |  |
| Number of cases | 29 | 24 | 20 | 22 |  |  |  |
| Model 2 | Ref | 0.94 (0.53,1.67) | 0.86 (0.45,1.63) | 1.01 (0.54,1.90) | 0.84 | 0.96 (0.85,1.10) | 0.57 |
| **Luminal breast cancer** |  |  |  |  |  |  |  |
| Number of cases | 87 | 92 | 107 | 81 |  |  |  |
| Model 2 | Ref | 1.16 (0.84,1.61) | 1.43 (1.02,1.99) | 1.15 (0.80,1.65) | 0.85 | 1.03 (0.97,1.10) | 0.31 |
| **Triple negative BC** |  |  |  |  |  |  |  |
| Number of cases | 12 | 13 | 8 | 21 |  |  |  |
| Model 2 | Ref | 1.03 (0.47,2.30) | 0.62 (0.25,1.57) | 1.53 (0.68,3.46) | 0.15 | 1.08 (0.89,1.30) | 0.46 |
| **Breast cancer <55 yo** |  |  |  |  |  |  |  |
| Number of cases | 70 | 49 | 31 | 30 |  |  |  |
| Model 2 | Ref | 1.00 (0.66,1.51) | 0.87 (0.53,1.43) | 1.02 (0.61,1.70) | 0.95 | 0.98 (0.89,1.09) | 0.75 |
| **Breast cancer ≥55 yo** |  |  |  |  |  |  |  |
| Number of cases | 81 | 112 | 126 | 118 |  |  |  |
| Model 2 | Ref | 1.29 (0.93,1.78) | 1.36 (0.98,1.90) | 1.30 (0.92,1.85) | 0.47 | 1.04 (0.97,1.10) | 0.27 |
| **Low-grade Pca** |  |  |  |  |  |  |  |
| Number of cases | 83 | 91 | 115 | 90 |  |  |  |
| Model 2 | Ref | 1.11 (0.79,1.56) | 1.35 (0.97,1.86) | 1.13 (0.79,1.63) | 0.74 | 1.06 (0.98,1.14) | 0.12 |
| **High-grade Pca** |  |  |  |  |  |  |  |
| Number of cases | 41 | 40 | 42 | 36 |  |  |  |
| Model 2 | Ref | 0.93 (0.57,1.51) | 0.94 (0.59,1.51) | 0.83 (0.49,1.40) | 0.49 | 0.98 (0.88,1.09) | 0.73 |
| **Cystatin-C** | | | | | | | |
| **Breast cancer ER-** |  |  |  |  |  |  |  |
| Number of cases | 30 | 37 | 29 | 16 |  |  |  |
| Model 2 | Ref | 1.34 (0.77,2.31) | 1.08 (0.59,1.99) | 0.63 (0.30,1.33) | 0.10 | 0.89 (0.63,1.24) | 0.48 |
| **Breast cancer Her2+** |  |  |  |  |  |  |  |
| Number of cases | 26 | 27 | 22 | 20 |  |  |  |
| Model 2 | Ref | 1.28 (0.71,2.31) | 1.19 (0.63,2.22) | 1.26 (0.63,2.54) | 0.62 | 1.05 (0.77,1.44) | 0.76 |
| **Luminal breast cancer** |  |  |  |  |  |  |  |
| Number of cases | 110 | 85 | 82 | 89 |  |  |  |
| Model 2 | Ref | 0.73 (0.52,1.02) | 0.68 (0.49,0.96) | 0.77 (0.53,1.12) | 0.37 | 0.81 (0.67,0.98) | 0.03 |
| **Triple negative BC** |  |  |  |  |  |  |  |
| Number of cases | 6 | 24 | 16 | 7 |  |  |  |
| Model 2 | Ref | 4.22 (1.60,11.13) | 2.68 (0.90,7.93) | 1.24 (0.36,4.25) | 0.28 | 0.83 (0.58,1.17) | 0.29 |
| **Breast cancer <55 yo** |  |  |  |  |  |  |  |
| Number of cases | 91 | 46 | 27 | 18 |  |  |  |
| Model 2 | Ref | 0.82 (0.55,1.22) | 0.77 (0.47,1.25) | 1.08 (0.58,1.98) | 0.82 | 0.89 (0.67,1.18) | 0.41 |
| **Breast cancer ≥55 yo** |  |  |  |  |  |  |  |
| Number of cases | 81 | 116 | 114 | 125 |  |  |  |
| Model 2 | Ref | 1.04 (0.73,1.48) | 0.86 (0.60,1.24) | 0.89 (0.61,1.29) | 0.38 | 0.86 (0.73,1.02) | 0.08 |
| **Low-grade Pca** |  |  |  |  |  |  |  |
| Number of cases | 73 | 106 | 80 | 114 |  |  |  |
| Model 2 | Ref | 1.16 (0.83,1.61) | 0.79 (0.55,1.13) | 1.09 (0.78,1.53) | 0.87 | 1.05 (0.90,1.23) | 0.55 |
| **High-grade Pca** |  |  |  |  |  |  |  |
| Number of cases | 34 | 41 | 41 | 42 |  |  |  |
| Model 2 | Ref | 1.09 (0.68,1.74) | 0.95 (0.58,1.53) | 0.96 (0.59,1.55) | 0.72 | 1.00 (0.79,1.26) | 0.99 |

ER: Estrogen Receptor, BC: breast cancer, Pca = Prostate cancer.
Luminal breast cancers include ER+/Her2- tumours.
Quartiles are based on the sex-specific distribution of the biomarker in the subcohort.
P-heterogeneity tests for breast cancer subtypes, age at diagnosis, and prostate cancer grade were respectively:
GDF-15: 0.50, 0.72, **0.05**
NT-proBNP: 0.46, 0.07, 0.33
HbA1C: **0.04**, 0.38, 0.21
CRP: 0.62, 0.23, 0.54
Cystatin-C: 0.21, 0.43, 0.73
Model 2 is a cause-specific Cox model stratified for age (as 5-y categories), adjusted for age (timescale), sex, BMI, lifetime alcohol consumption, smoking status (never, long time quitters, short time quitters, current light, and current heavy smokers), physical activity level, educational level, and baseline self-reported diabetes (for GDF-15 and HbA1C).
Continuous HR for one unit increment in log-2 based biomarker = change in hazard associated with a doubling of biomarker concentration
HRs were corrected to match case-cohort design using inverse subcohort sampling probability weighting (ISSP)

**Table S3 - Hazard ratios and 95%CI for associations between GDF-15, NT-proBNP, HBA1C, CRP and cystatin-C with risks of cancer and cardiovascular diseases, after exclusion of cases diagnosed within the first 2 years of follow-up, EPIC-Heidelberg case-cohort**

|  | **Q1** | **Q2** | **Q3** | **Q4** | **P-trend** | **Continuous** | **P** |  |
| --- | --- | --- | --- | --- | --- | --- | --- | --- |
|  |  |  |  |  |  |  |  |  |
| **GDF-15** | | | | | | | | |
| **Breast cancer** |  |  |  |  |  |  |  |  |
| Number of cases | 132 | 164 | 138 | 133 |  |  |  |  |
| Model 2 | Ref | 1.26 (0.96,1.66) | 1.16 (0.86,1.56) | 1.21 (0.89,1.65) | 0.42 | 1.03 (0.89,1.18) | 0.71 |  |
| **Prostate cancer** |  |  |  |  |  |  |  |  |
| Number of cases | 127 | 135 | 154 | 123 |  |  |  |  |
| Model 2 | Ref | 0.85 (0.64,1.13) | 0.83 (0.62,1.11) | 0.69 (0.50,0.95) | 0.03 | 0.75 (0.59,0.94) | 0.04 |  |
| **Lung cancer** |  |  |  |  |  |  |  |  |
| Number of cases | 19 | 28 | 44 | 107 |  |  |  |  |
| Model 2 | Ref | 1.09 (0.59,2.02) | 1.23 (0.68,2.25) | 2.64 (1.51,4.62) | <.001 | 1.60 (1.33,1.91) | <.001 |  |
| **Colorectal cancer** |  |  |  |  |  |  |  |  |
| Number of cases | 42 | 69 | 66 | 66 |  |  |  |  |
| Model 2 | Ref | 1.30 (0.86,1.97) | 1.04 (0.67,1.61) | 0.99 (0.62,1.58) | 0.52 | 0.84 (0.65,1.10) | 0.21 |  |
| **Mycordial Infarction** |  |  |  |  |  |  |  |  |
| Number of cases | 102 | 131 | 206 | 222 |  |  |  |  |
| Model 2 | Ref | 1.05 (0.74,1.40) | 1.39 (1.05,1.84) | 1.40 (1.04,1.88) | 0.03 | 1.34 (1.14,1.56) | <.001 |  |
| **Stroke** |  |  |  |  |  |  |  |  |
| Number of cases | 94 | 134 | 186 | 270 |  |  |  |  |
| Model 2 | Ref | 1.18 (0.88,1.58) | 1.39 (1.04,1.85) | 1.95 (1.45,2.64) | <.001 | 1.47 (1.28,1.69) | <.001 |  |
| **NT-proBNP** | | | | | | | | |
| **Breast cancer** |  |  |  |  |  |  |  |  |
| Number of cases | 91 | 150 | 150 | 156 |  |  |  |  |
| Model 2 | Ref | 1.69 (1.26,2.28) | 1.71 (1.26,2.32) | 1.79 (1.33,2.42) | 0.002 | 1.17 (1.09,1.26) | <.001 |  |
| **Prostate cancer** |  |  |  |  |  |  |  |  |
| Number of cases | 87 | 119 | 142 | 165 |  |  |  |  |
| Model 2 | Ref | 1.41 (1.03,1.93) | 1.73 (1.27,2.35) | 1.84 (1.36,2.48) | <.001 | 1.17 (1.09,1.25) | <.001 |  |
| **Lung cancer** |  |  |  |  |  |  |  |  |
| Number of cases | 37 | 50 | 54 | 46 |  |  |  |  |
| Model 2 | Ref | 1.34 (0.84,2.13) | 1.49 (0.94,2.37) | 1.18 (0.74,1.89) | 0.95 | 1.08 (0.97,1.20) | 0.16 |  |
| **Colorectal cancer** |  |  |  |  |  |  |  |  |
| Number of cases | 40 | 63 | 48 | 81 |  |  |  |  |
| Model 2 | Ref | 1.65 (1.09,2.52) | 1.29 (0.83,2.01) | 2.05 (1.37,3.06) | <.001 | 1.16 (1.06,1.26) | <.001 |  |
| **Mycordial Infarction** |  |  |  |  |  |  |  |  |
| Number of cases | 130 | 142 | 164 | 190 |  |  |  |  |
| Model 2 | Ref | 1.13 (0.86,1.49) | 1.32 (1.01,1.73) | 1.55 (1.20,2.01) | <.001 | 1.15 (1.08,1.23) | <.001 |  |
| **Stroke** |  |  |  |  |  |  |  |  |
| Number of cases | 141 | 135 | 168 | 210 |  |  |  |  |
| Model 2 | Ref | 0.98 (0.76,1.28) | 1.19 (0.92,1.54) | 1.39 (1.09,1.78) | 0.001 | 1.13 (1.06,1.21) | <.001 |  |
| **HbA1C** | | | | | | | | |
| **Breast cancer** |  |  |  |  |  |  |  |  |
| Number of cases | 163 | 139 | 172 | 140 |  |  |  |  |
| Model 2 | Ref | 1.33 (1.03,1.73) | 1.36 (1.05,1.75) | 1.38 (1.04,1.84) | 0.03 | 1.63 (0.98,2.71) | 0.06 |  |
| **Prostate cancer** |  |  |  |  |  |  |  |  |
| Number of cases | 169 | 97 | 144 | 137 |  |  |  |  |
| Model 2 | Ref | 1.00 (0.75,1.34) | 1.24 (0.95,1.61) | 1.19 (0.89,1.59) | 0.14 | 1.32 (0.86,2.02) | 0.20 |  |
| **Lung cancer** |  |  |  |  |  |  |  |  |
| Number of cases | 36 | 31 | 66 | 74 |  |  |  |  |
| Model 2 | Ref | 1.19 (0.72,1.99) | 1.89 (1.19,3.00) | 1.75 (1.07,2.84) | 0.02 | 2.70 (1.32,5.53) | 0.01 |  |
| **Colorectal cancer** |  |  |  |  |  |  |  |  |
| Number of cases | 68 | 41 | 68 | 78 |  |  |  |  |
| Model 2 | Ref | 0.94 (0.62,1.40) | 1.21 (0.84,1.73) | 1.16 (0.78,1.74) | 0.32 | 1.21 (0.67,2.17) | 0.53 |  |
| **Mycordial Infarction** |  |  |  |  |  |  |  |  |
| Number of cases | 157 | 104 | 171 | 248 |  |  |  |  |
| Model 2 | Ref | 1.01 (0.77,1.34) | 1.33 (1.04,1.71) | 1.48 (1.14,1.92) | 0.00 | 1.90 (1.25,2.89) | <.001 |  |
| **Stroke** |  |  |  |  |  |  |  |  |
| Number of cases | 169 | 123 | 173 | 211 |  |  |  |  |
| Model 2 | Ref | 1.11 (0.86,1.43) | 1.22 (0.96,1.55) | 1.20 (0.92,1.56) | 0.20 | 1.35 (0.89,2.04) | 0.16 |  |
| **CRP** | | | | | | | | |
| **Breast cancer** |  |  |  |  |  |  |  |  |
| Number of cases | 139 | 147 | 147 | 130 |  |  |  |  |
| Model 2 | Ref | 1.16 (0.89,1.52) | 1.22 (0.92,1.61) | 1.13 (0.84,1.52) | 0.79 | 1.02 (0.96,1.08) | 0.52 |  |
| **Prostate cancer** |  |  |  |  |  |  |  |  |
| Number of cases | 124 | 130 | 153 | 128 |  |  |  |  |
| Model 2 | Ref | 1.04 (0.78,1.40) | 1.18 (0.89,1.56) | 1.03 (0.75,1.42) | 0.99 | 1.03 (0.97,1.10) | 0.30 |  |
| **Lung cancer** |  |  |  |  |  |  |  |  |
| Number of cases | 23 | 37 | 52 | 81 |  |  |  |  |
| Model 2 | Ref | 1.37 (0.78,2.42) | 1.67 (0.97,2.86) | 2.15 (1.25,3.71) | 0.01 | 1.15 (1.04,1.26) | 0.01 |  |
| **Colorectal cancer** |  |  |  |  |  |  |  |  |
| Number of cases | 41 | 55 | 71 | 68 |  |  |  |  |
| Model 2 | Ref | 1.16 (0.75,1.78) | 1.34 (0.88,2.05) | 1.22 (0.77,1.94) | 0.63 | 1.05 (0.96,1.15) | 0.33 |  |
| **Mycordial Infarction** |  |  |  |  |  |  |  |  |
| Number of cases | 86 | 117 | 187 | 261 |  |  |  |  |
| Model 2 | Ref | 1.14 (0.84,1.56) | 1.66 (1.24,2.23) | 2.09 (1.55,2.82) | <.001 | 1.18 (1.12,1.25) | <.001 |  |
| **Stroke** |  |  |  |  |  |  |  |  |
| Number of cases | 98 | 136 | 190 | 249 |  |  |  |  |
| Model 2 | Ref | 1.23 (0.93,1.64) | 1.55 (1.18,2.04) | 1.93 (1.46,2.56) | <.001 | 1.14 (1.08,1.20) | <.001 |  |
| **Cystatin-C** | | | | | | | | |
| **Breast cancer** |  |  |  |  |  |  |  |  |
| Number of cases | 162 | 146 | 129 | 125 |  |  |  |  |
| Model 2 | Ref | 0.92 (0.70,1.21) | 0.81 (0.60,1.09) | 0.83 (0.61,1.14) | 0.27 | 0.85 (0.73,0.99) | 0.04 |  |
| **Prostate cancer** |  |  |  |  |  |  |  |  |
| Number of cases | 107 | 147 | 122 | 150 |  |  |  |  |
| Model 2 | Ref | 1.13 (0.85,1.50) | 0.83 (0.62,1.13) | 1.00 (0.75,1.34) | 0.67 | 1.01 (0.88,1.16) | 0.91 |  |
| **Lung cancer** |  |  |  |  |  |  |  |  |
| Number of cases | 43 | 46 | 58 | 47 |  |  |  |  |
| Model 2 | Ref | 1.04 (0.64,1.67) | 1.21 (0.76,1.92) | 1.00 (0.61,1.64) | 0.99 | 1.06 (0.86,1.31) | 0.59 |  |
| **Colorectal cancer** |  |  |  |  |  |  |  |  |
| Number of cases | 50 | 59 | 61 | 69 |  |  |  |  |
| Model 2 | Ref | 1.02 (0.68,1.51) | 0.99 (0.66,1.48) | 1.05 (0.70,1.55) | 0.81 | 1.02 (0.85,1.23) | 0.83 |  |
| **Mycordial Infarction** |  |  |  |  |  |  |  |  |
| Number of cases | 139 | 161 | 151 | 198 |  |  |  |  |
| Model 2 | Ref | 1.10 (0.85,1.43) | 0.99 (0.75,1.29) | 1.22 (0.94,1.59) | 0.15 | 1.17 (1.03,1.32) | 0.02 |  |
| **Stroke** |  |  |  |  |  |  |  |  |
| Number of cases | 125 | 171 | 195 | 187 |  |  |  |  |
| Model 2 | Ref | 1.17 (0.91,1.52) | 1.27 (0.98,1.65) | 1.15 (0.88,1.49) | 0.57 | 1.12 (0.99,1.26) | 0.07 |  |

Model 2 is a cause-specific Cox model stratified for age (as 5-y categories), adjusted for age (timescale), sex, BMI, lifetime alcohol consumption, smoking status (never, long time quitters, short time quitters, current light, and current heavy smokers), physical activity level, educational level, baseline self-reported diabetes (for GDF-15 and HbA1C), and baseline self-reported hypertension (for myocardial infarction and stroke).
Continuous HR for one unit increment in log-2 based biomarker = change in hazard associated with a doubling of biomarker concentration
HRs were corrected to match case-cohort design using inverse sub-cohort sampling probability weighting (ISSP)
